# Oncogenic Activity and Sorafenib Sensitivity of *ARAF* p.S214C Mutation in Lung Cancer

**DOI:** 10.3390/cancers17132246

**Published:** 2025-07-04

**Authors:** Carol Lee, Weixue Mu, Xi July Chen, Mandy Sze Man Chan, Zhishan Chen, Sai Fung Yeung, Helen Hoi Yin Chan, Sin Ting Chow, Ben Chi Bun Ko, David Wai Chan, William C. Cho, Vivian Wai Yan Lui, Stephen Kwok Wing Tsui

**Affiliations:** 1 School of Biomedical Sciences, The Chinese University of Hong Kong, Hong Kong; carollee@cuhk.edu.hk (C.L.); muweixue@link.cuhk.edu.hk (W.M.); julychen@cuhk.edu.hk (X.J.C.); 22040547r@connect.polyu.hk (M.S.M.C.); 1155170904@link.cuhk.edu.hk (Z.C.); sfrk.yeung@cuhk.edu.hk (S.F.Y.); helenchanhy@link.cuhk.edu.hk (H.H.Y.C.); cindychowsinting@yahoo.com (S.T.C.); 2 Department of Applied Biology and Chemical Technology, The Hong Kong Polytechnic University, Hong Kong; ben.ko@polyu.edu.hk; 3 School of Medicine, The Chinese University of Hong Kong, Shenzhen 518172, China; dwchan@cuhk.edu.cn; 4 Department of Clinical Oncology, Queen Elizabeth Hospital, Hong Kong; williamcscho@gmail.com; 5 Georgia Cancer Center, Department of Biochemistry and Molecular Biology, Medical College of Georgia, Augusta University, Augusta, GA 30912, USA

**Keywords:** *ARAF* p.S214C mutation, oncogenicity, sorafenib sensitivity, lung cancer

## Abstract

**Background/Objectives**: RAF pathway aberrations are one of the hallmarks of lung cancer. Sorafenib is a multi-kinase inhibitor targeting the RAF pathway and is FDA-approved for several cancers, yet its efficacy in lung cancer is controversial. Previous clinical research showed that a *ARAF* p.S214C mutation exhibited exceptional responsiveness to sorafenib in lung adenocarcinoma. **Methods**: Considering this promising clinical potential, the oncogenic potential and sorafenib response of the *ARAF* p.S214C mutation were investigated using lung cancer models. *ARAF* p.S214C mutant, *ARAF* wild-type (WT), and *EGFP* control genes were ectopically expressed in lung adenocarcinoma cell lines retroviral transduction. In vitro and in vivo sorafenib sensitivity studies were performed, followed by transcriptomics and proteomics analyses. **Results**: Compared to the *ARAF*-WT and *EGFP*-engineered cells, the *ARAF* p.S214C-engineered cells activated Raf-MEK-ERK signaling and exhibited enhanced oncogenic potential in terms of in vitro cell proliferation, colony and spheroid formation, migration, and invasion abilities, as well as in vivo tumorigenicity. The *ARAF* p.S214C-engineered cells also displayed heightened sensitivity to sorafenib in vitro and in vivo. RNA sequencing and reverse-phase protein array analyses demonstrated elevated expression of genes and proteins associated with tumor aggressiveness in the *ARAF* p.S214C mutants, and its sorafenib sensitivity was likely moderated through inhibition of the cell cycle and DNA replication. The ERK and PI3K signaling pathways were also significantly deregulated in the *ARAF* p.S214C mutants regardless of sorafenib treatment. **Conclusions**: This study demonstrates the oncogenicity and sorafenib sensitivity of the *ARAF* p.S214C mutation in lung cancer cells, which may serve as a biomarker for predicting the sorafenib response in lung cancer patients. Importantly, investigating the gene–drug sensitivity pairs in clinically exceptional responders may guide and accelerate personalized cancer therapies based on specific tumor mutations.

## Simple Summary

Lung cancer is often associated with aberrated signaling pathways like the RAF pathway. While most studies have focused on *BRAF* and *CRAF* mutations, the role of *ARAF* is less understood. This study shows that *ARAF* p.S214C mutation is a rare but important mutation that exhibits oncogenic properties in lung cancer models. *ARAF* p.S214C-mutated lung cancer cells demonstrate remarkable sorafenib sensitivity in vitro and in vivo, which aligns with a previous clinical report of a lung cancer patient carrying this mutation being an exceptional responder to sorafenib. These findings provide strong evidence that *ARAF* p.S214C mutation may serve as a novel biomarker for predicting sorafenib efficacy in lung cancer. Investigating the gene–drug sensitivity pairs in clinically exceptional responders in non-precision-based clinical trials could inform personalized cancer treatment strategies, leading to more effective and faster approval of targeted therapies based on specific tumor mutations.

## 1. Introduction

Lung cancer is the leading cause of cancer mortality worldwide, with adenocarcinoma as the predominant subtype (~40%) [1]. *KRAS* mutations are the most common mutations in lung cancer (~30%) [2]. Despite recent advancements in KRAS-targeted therapies, frequent resistance to KRAS inhibitors necessitates continuous efforts to target downstream effectors, particularly RAF kinases [3]. The RAF family of serine/threonine kinases comprises three isoforms: ARAF, BRAF, and CRAF. They share three conserved regions (CRs): CR1, which contains the RAS-binding domain (RBD) and the phorbol ester/diacylglycerol-binding (C1) domain; CR2, the regulatory domain; and CR3, the protein kinase domain. RAF proteins are activated by binding to activated RAS proteins, leading to their homo- and heterodimerization, which then activates the mitogen-activated protein kinase (MEK)–extracellular-signal-regulated kinase (ERK) signaling cascade [4]. The Raf-MEK-ERK pathway is crucial for cell proliferation and survival, and aberrations of this pathway have been implicated in oncogenesis [5,6]. *BRAF* mutations are the most prevalent among the three RAF isoforms, occurring in 1.5–3.5% of lung cancer cases [7,8]. While most studies have focused on BRAF and CRAF, the pathological significance of ARAF in lung cancer remains unclear.

To date, one effective clinically approved inhibitor of RAF activity is sorafenib, it is also recognized as a multi-kinase inhibitor that targets vascular endothelial growth factor receptor (VEGFR), platelet-derived growth factor receptor (PDGFR), c-Kit, and the rearranged during transfection (RET) oncoprotein [9]. Sorafenib is FDA-approved for treating hepatocellular carcinoma, renal cell carcinoma, and thyroid cancer [10,11,12], yet its effectiveness in lung cancer has been elusive due to the heterogeneity of the disease [13]. Understanding the molecular landscape and identifying biomarkers associated with sorafenib responsiveness are crucial to optimizing the therapeutic outcomes in lung cancer. Importantly, one clinical study reported an exceptional response to sorafenib with near-complete tumor regression in a lung adenocarcinoma patient carrying an *ARAF* p.S214C mutation [14], highlighting the potential of *ARAF* mutations in contributing to sorafenib sensitivity in lung cancer. Given this promising clinical potential, the present study aimed to investigate the oncogenic potential and sorafenib response of *ARAF* p.S214C mutation in lung cancer further.

Here, using lung adenocarcinoma cell lines and animal models, it is demonstrated that the *ARAF* p.S214C mutation is associated with enhanced oncogenic signaling and sensitivity to sorafenib. By characterizing the gene–drug sensitivity pair of *ARAF* p.S214C and sorafenib, this study may help establish *ARAF* p.S214C mutation as a novel biomarker for predicting sorafenib efficacy, which could implicate a new precision medicine strategy for lung cancer.

## 2. Materials and Methods

### 2.1. Resources

A list of the resources used in this study is available in Table S1.

### 2.2. Mutation Database Acquisition

Mutation data for ARAF were downloaded from the American Association for Cancer Research (AACR) Project Genomics Evidence Neoplasia Information Exchange (GENIE) public database cohort v14.1 (https://www.aacr.org/professionals/research/aacr-project-genie/aacr-project-genie-data/ (accessed on 5 November 2024)). Data from a pan-cancer cohort, a pan-lung cancer cohort, and a lung adenocarcinoma cohort from multiple centers were retrieved for analysis.

### 2.3. The Cell Cultures and Drugs

The human lung adenocarcinoma cell lines NCI-H2023 and NCI-H522 were purchased from the American Type Culture Collection (ATCC), Manassas, VA, USA and cultured in RPMI 1640 with 10% fetal bovine serum (FBS), 1% penicillin/streptomycin (P/S), and 1% sodium pyruvate (Gibco, Grand Island, NY, USA). Note that in all the figures, the names of these cell lines were abbreviated as H2023 and H522, respectively. The Platinum-A (PLAT-A) retrovirus packaging cell line was purchased from Cell Biolabs (San Diego, CA, USA) and cultured in DMEM with 10% FBS, 1% P/S, 10 µg/mL of blasticidin (Gibco, USA), and 1 µg/mL of puromycin (Sigma-Aldrich, St. Louis, MO, USA). The cells were maintained at 37 °C under 5% CO_2_, confirmed to be mycoplasma-free using the VenorGeM OneStep Mycoplasma detection kit (Minerva Biolabs, Berlin, Germany), and authenticated through short tandem repeat (STR) analyses (Pangenia, Toronto, ON, Canada). Results of the cell line authentication can be found in Supplementary Materials. Sorafenib (MedChemExpress, Princeton, NJ, USA) and PB98059 (Selleckchem, Houston, TX, USA) were prepared in DMSO (vehicle, Sigma-Aldrich, USA).

### 2.4. Retroviral Vectors and Infection

The pMXs-puro and pMXs-EGFP-puro retroviral vectors were purchased from Cell Biolabs, USA. *ARAF*-WT and *ARAF* p.S214C were cloned into pMXs-puro. The vectors were transfected into the PLAT-A cells using Lipofectamine 3000 (Invitrogen, Carlsbad, CA, USA) for 72 h. Retroviruses were filtered through a 0.45 µm polyethersulfone membrane to infect the NCI-H2023 and NCI-H522 cells for 72 h. Cells were selected with 2 µg/mL of puromycin for 7 days.

### 2.5. Western Blotting

Whole-cell lysates were prepared in Nonidet P-40 lysis buffer, centrifuged, and quantified using Protein Assay Dye Reagent (Bio-Rad, Hercules, CA, USA). A total of 10 μg of the proteins was subjected to SDS-PAGE and transferred onto nitrocellulose membranes. The membranes were blocked with 3% bovine serum albumin and probed with the primary antibody at 4 °C overnight, followed by incubation with HRP-conjugated secondary antibodies for 1 h. The proteins were visualized using an enhanced chemiluminescence (ECL) detection kit (Thermo Fisher Scientific, Waltham, MA, USA). The antibodies used for Western blotting are listed in Table S1.

### 2.6. The MTT Assay

The NCI-H2023 and NCI-H522 cells were seeded into 96-well plates at 3000 or 6000 cells/100 µL/well, respectively, in RPMI with 1% FBS with or without drug treatment. The cell viability was determined using the 3-(4,5-dimethyl-2-thiazolyl)-2,5-diphenyl-2H-tetrazolium bromide (MTT) assay (Invitrogen, USA), with the absorbance measured at 570 nm. To assess the oncogenic potential, the cells were grown for 10 days in the absence of drugs, and the cell viability was determined daily. To assess the drug response, the cells were treated with the DMSO control or sorafenib (NCI-H2023: 0.25 µM, 0.5 µM, 1 µM, 2 µM, 4 µM, 8 µM, 16 µM, 32 µM, and 64 µM; NCI-H522: 0.5 µM, 1 µM, 2 µM, 4 µM, 6 µM, 8 µM, 12 µM, 16 µM, and 32 µM) or PD98059 (NCI-H2023 and NCI-H522: 0.25 µM, 1 µM, 10 µM, 25 µM, 50 µM, 75 µM, 100 µM, 150 µM, 250 µM, 400 µM, and 500 µM) for 72 h, and the cell viability was determined at the experimental endpoint.

### 2.7. The Migration Assay

The NCI-H2023 and NCI-H522 cells were seeded into 2-well culture inserts with a gap width of 500 µm (ibidi, Munich, Germany) at 4 × 10^4^ cells/100 µL/insert and 5 × 10^4^ cells/100 µL/insert, respectively, in RPMI with 1% FBS. On the next day, the inserts were removed, and the cells were replenished with RPMI with 10% FBS. After 24 h or 48 h, the area of gap closure for the NCI-H2023 and NCI-H522 cells was quantified, respectively, using ImageJ (v.1.54h; RRID: SCR_003070). Brightfield images were taken using a light microscope.

### 2.8. The Invasion Assay

Transwell chambers with 8 µm pore membranes (SPL Life Sciences, Pocheon, Republic of Korea) were inserted into 24-well plates. The cells were seeded into the upper chamber, pre-coated with Matrigel (Corning, Corning, NY, USA), at 4 × 10^4^ cells/200 µL/well in serum-free RPMI medium. The lower chamber was loaded with 600 µL of RPMI medium with 10% FBS. After 18 h and 48 h, the NCI-H2023 and NCI-H522 cells in the upper chamber were removed, respectively. Invaded cells were fixed in 4% paraformaldehyde and stained using crystal violet. Brightfield images were taken with a light microscope. Ten random fields of view were quantified at 100× magnification per well using ImageJ.

### 2.9. The Colony Formation Assay

Cells were seeded into 6-well plates at 1000 cells/2 mL/well in RPMI medium with 10% FBS with or without sorafenib treatment. The NCI-H2023 and NCI-H522 colonies were grown for 7 and 14 days, respectively; fixed in 4% paraformaldehyde; and stained using crystal violet. To assess the oncogenic potential, the colonies were grown in the absence of drugs. To assess the sorafenib response, the colonies were treated with the DMSO control or sorafenib (NCI-H2023: 2 µM, 4 µM; NCI-H522: 1 µM, 2 µM). The number of colonies with a diameter of over 250 µm was quantified using ImageJ.

### 2.10. The Spheroid Formation Assay

To assess the oncogenic potential, the NCI-H2023 and NCI-H522 cells were seeded into 96-well, round-bottom, ultra-low-attachment plates at 1000 cells/100 µL/well and 5000 cells/100 µL/well, respectively, in RPMI medium with 10% FBS. Spheroids were grown for 10 days in the absence of drugs. Spheroid growth was measured in terms of the spheroid area using ImageJ, and spheroid cell viability was assessed using the CellTiter-Glo 3D Cell Viability Assay (Promega, Madison, WI, USA) according to the manufacturer’s instructions. To assess the sorafenib response, both cell lines were seeded into 96-well, round-bottom, ultra-low-attachment plates at 1000 cells/100 µL/well in RPMI medium with 10% FBS. Spheroids were grown for 4 days in the absence of drugs and then treated with the DMSO control or sorafenib (NCI-H2023: 8 µM, 16 µM; H522: 4 µM, 8 µM) for 72 h. The CellTiter-Glo Luminescent Cell Viability Assay (Promega, USA) and Hoechst 33342/propidium iodide (Invitrogen, USA) staining were performed according to the manufacturer’s instructions to determine the spheroid cells’ viability and apoptotic rate, respectively. Images were taken using the CELENA X High Content Imaging System (Logos Biosystems, Anyang, Republic of Korea).

### 2.11. RNA Sequencing

NCI-H2023 cells expressing *EGFP*, *ARAF*-WT, and *ARAF* p.S214C were treated with DMSO or 4 µM of sorafenib for 72 h. RNA was extracted using the RNeasy Mini Kit (Qiagen, Hilden, Germany) and then reversed-transcribed using the High-Capacity cDNA Reverse Transcription Kit (Applied Biosystems, Foster City, CA, USA). The RNA (5 µg per sample) was sent for RNA sequencing (RNA-Seq, Illumina HiSeq platform, Novogene HK, Hong Kong). The raw RNA-Seq data were trimmed using Trimmomatic (v0.39) to remove low-quality bases and adapters. High-quality reads were mapped to the human reference genome (GRCh38) using Hisat2 (v2.2.1; RRID: SCR_015530). The transcript counts for the gene expression levels were calculated using FeatureCounts (v2.0.2; RRID: SCR_012919), and the relative abundance was determined as the FPKM using Stringtie (v2.2.1; RRID: SCR_016323). The differentially expressed gene (DEG) analysis was conducted using DESeq2 (v3.18; RRID: SCR_015687), with a cutoff of log2(fold-change) ≤ −1 or ≥1 and an adjusted *p* < 0.05. The online tool DAVID (http://david-d.ncifcrf.gov/) was used for the Gene Ontology (GO) and Kyoto Encyclopedia of Genes and Genomes (KEGG) pathway enrichment analysis of the DEGs. Heatmaps of selected DEGs were generated using Hiplot (https://hiplot.cn). The FPKM in each row was normalized using the z-score, and both rows and columns were clustered.

### 2.12. The Reverse-Phase Protein Array Analysis

The H2023 cells expressing *EGFP*, *ARAF*-WT, and *ARAF* p.S214C were treated with DMSO or 4 µM of sorafenib for 72 h. These samples were derived from the same batch as that used for RNA sequencing, as described in Section 2.11. Protein lysates (>150 µg per sample) were prepared as described in Section 2.5 and sent for the reverse-phase protein array analysis (RPPA, DaHong Biosciences, Guangzhou, China). Approximately 400 identical slides were quantified in terms of the colorimetric signals of 384 antibodies (Table S9). All RPPA data were processed using R (v4.1.0). The raw RPPA data underwent quality control and normalization through median centering across all antibodies (Table S10), followed by log2 transformation to generate an expression matrix (Table S11). Data cleaning and clustering, a differential expression analysis, a correlation analysis, and an overlapping analysis were performed and plotted mainly using R packages (dplyr, ggplot2, pheatmap, clusterProfiler, and VennDiagram; v4.3.2; RRID: SCR_014601). The Wilcoxon ranked sum test was employed for the analysis of the differential expression of individual RPPA targets, with a cutoff of log2(fold-change) ≤ −1 or ≥1 and *p* < 0.05.

### 2.13. The Animal Experiments

The animal experiments were approved by the Animal Experimentation Ethics Committee of the Chinese University of Hong Kong (CUHK; reference number: RIF-19-198-4B; date of approval: 23 Aug 2019; consent number: 19-198-RIF). Four- to six-week-old male Nu/J mice were used, which were bred from the Laboratory Animal Services Centre, CUHK. The H2023 cells expressing *EGFP*, *ARAF*-WT, and *ARAF* p.S214C were subcutaneously injected into the left and right flanks of the mice at 2 × 10^6^ cells in HBSS per tumor, respectively. After 7 days of tumor inoculation, sorafenib (10 mg/kg and 20 mg/kg) or the vehicle (cremophor EL/95% ethanol) was administered daily via oral gavage for 18 days, with each group comprising 4 mice, each bearing 2 tumors. The tumor volume was monitored and calculated as (length × width^2^)/2. At the experimental endpoint, the tumors were harvested and formalin-fixed.

### 2.14. Immunohistochemistry and Hematoxylin and Eosin Staining

The formalin-fixed tumors were embedded in paraffin. Immunohistochemistry (IHC) of the tumor sections was performed using the VECTASTAIN Elite ABC-HRP Kit, Peroxidase (VectorLabs, Burlingame, CA, USA), according to the manufacturer’s instructions. Tissues were counterstained with hematoxylin, dehydrated, and mounted with a coverslip. For hematoxylin and eosin (H&E) staining, the deparaffinized and rehydrated sections were sequentially dipped in Harris hematoxylin, 1% acid alcohol, and 1% eosin before dehydration and mounting. Images were taken using an Axioscan 7 Automatic Microscope Slide Scanner (ZEISS, Jena, Germany). The antibodies for IHC are listed in Table S1.

### 2.15. The Statistical Analysis

The statistical analysis was performed using GraphPad Prism (v9.5.1; serial number: GPS-2505185-TIT3-F100A; RRID: SCR_002798). Data were presented as the mean ± SD and considered significant at *p* < 0.05. An analysis of variance (ANOVA) with Tukey’s post hoc test was performed to compare groups. For clarity, the *p*-values reported in the text only compare the wild-type and mutant groups. The *p*-values from the pairwise statistical comparisons among *EGFP* control vector, wild-type, and mutant groups are detailed in Table S2.

## 3. Results

### 3.1. ARAF p.S214C Is a Rare but Important Mutation in Lung Adenocarcinoma

**Figure 1:**
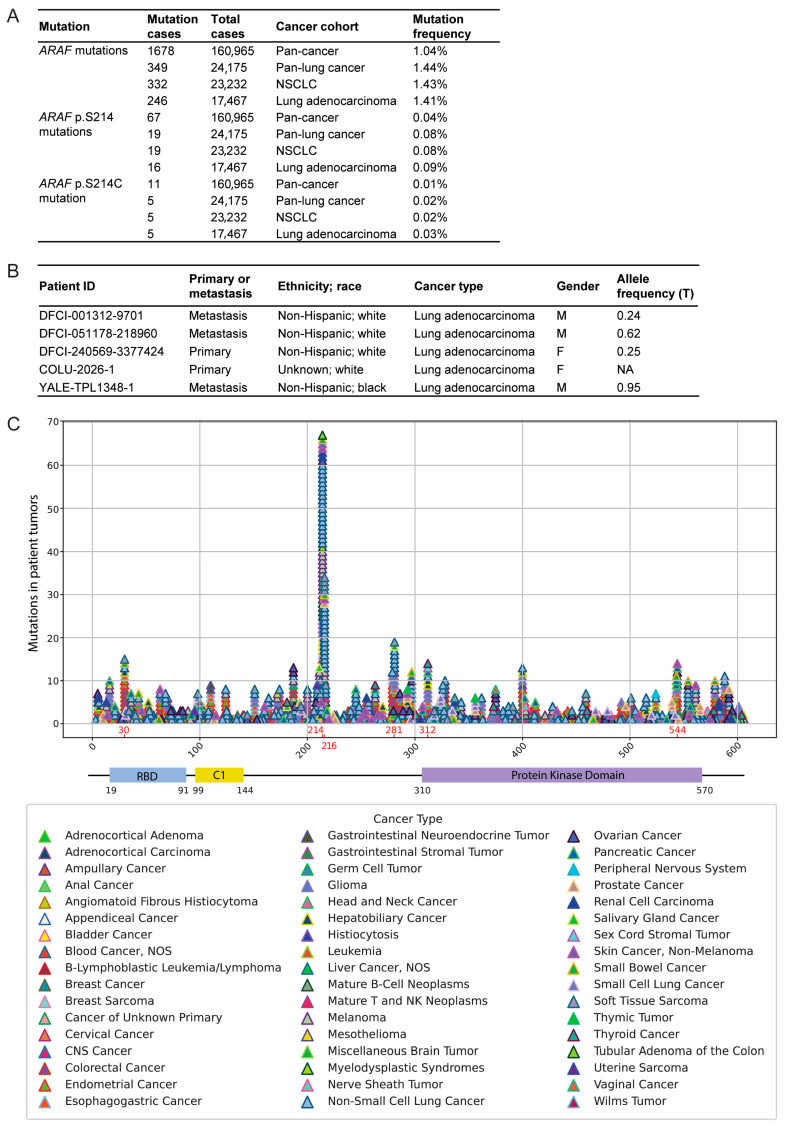
The *ARAF* mutation landscape in cancer. (**A**) The number and frequency of *ARAF* mutations in different cancer types in AACR Project GENIE public database cohort v14.1. (**B**) Lung cancer cases with *ARAF* p.S214C mutations in the GENIE public database cohort v14.1. All cases are adenocarcinoma. (**C**) Mapping of the *ARAF* mutation sites based on the pan-cancer data from GENIE database v14.1. Each mutation case is shown as one triangular symbol. Each cancer type is color-coded as shown. C1, phorbol ester/diacylglycerol-binding domain. RBD, Ras-binding domain.

In the AACR Project GENIE public database cohort v14.1, as depicted in Figure 1A, the *ARAF* mutation rate is 1.04% in the pan-cancer group (1678/160,965 cases), with pan-lung cancers displaying slightly elevated frequencies of ~1.44% (349/24,175 cases). Compared to the pan-cancer and pan-lung cancer cohorts, lung adenocarcinoma exhibits the highest mutation frequency at the *ARAF* p.S214 site, occurring at a rate of 0.09% (16/17,467 cases), and specifically at the p.S214C site, with a frequency of 0.03% (5/17,467 cases). Notably, all *ARAF* p.S214 mutations in lung cancer occur in adenocarcinoma, underscoring the rarity yet the significance of this mutation in the most prevalent subtype of lung cancer. The demographic and tumor characteristics of the five cases of *ARAF* p.S214C in the lung adenocarcinoma cohort are detailed in Figure 1B. The *ARAF* p.S214C mutation in these cases is characterized by the HGVSg notion 23:g.47426121C>G (genomic DNA) and the HGVSc notion ENST00000377045.4:c.641C>G (coding DNA). Interestingly, two patients bearing metastatic lung adenocarcinoma displayed elevated allele frequencies of 0.62 and 0.95, respectively, suggesting the potential role of *ARAF* p.S214C in driving tumor aggressiveness and metastatic activity.

Mapping all of the *ARAF* mutations from the pan-cancer cohort highlights that the most prominent clustering occurs at position S214 (67 cases), followed by the adjacent position P216 (34 cases, Figure 1C). Both mutation hotspots are located within the CR2 regulatory domain of *ARAF*, characterized by a serine/threonine-rich sequence where 14-3-3 binds and inhibits Raf, indicating that mutations at this region are likely to disrupt this inhibition, thereby activating Raf.

### 3.2. ARAF p.S214C Activates the MEK-ERK Pathway and Enhances the Proliferation, Colony and Spheroid Formation, Migration, and Invasion of Cells In Vitro

**Figure 2:**
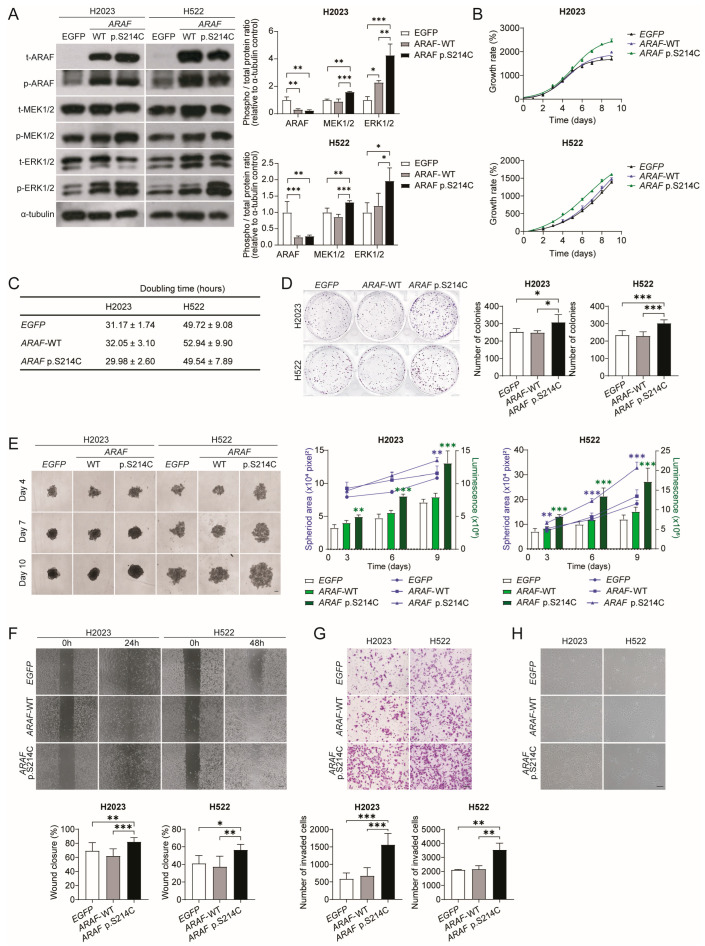
*ARAF* p.S214C activates the MEK/ERK pathway and enhances oncogenicity. (**A**) Western blot showing the expression levels of total (t)-ARAF, phospho-(p)-ARAF, t-MEK1/2, p-MEK1/2, t-ERK1/2, and p-ERK1/2 in H2023 and H522 cells ectopically expressing *EGFP*, *ARAF*-WT, and *ARAF* p.S214C, respectively (denoted as sublines below). α-tubulin was included as a loading control. Densitometric quantification of the phosphorylated and total expression of ARAF, MEK1/2, and ERK1/2 is shown in bar graphs. (**B**) The growth rate and (**C**) the doubling time of the H2023 and H522 sublines assessed through the MTT assay (H2023, *p* = 0.0144; H522, *p* = 0.0014). (**D**) Colony formation of H2023 (1000 cells/well) and H522 (1000 cells/well) sublines for 7 and 14 days, respectively (H2023, *p* = 0.016; H522, *p* < 0.001). The number of colonies with a diameter of over 250 µm was quantified using ImageJ. (**E**) Spheroid growth of the H2023 (1000 cells/well) and H522 (5000 cells/well) sublines on days 4, 7, and 10 in terms of the spheroid area measured using ImageJ (H2023, *p* = 0.0040; H522, *p* < 0.001) and the spheroid cell viability determined through the luminescence signal in the CellTiter-Glo 3D Cell Viability assay (H2023, *p* < 0.001; H522, *p* < 0.001). Scale bar, 200 μm. (**F**) The migration of the H2023 (4 × 10^4^ cells/insert) and H522 (5 × 10^4^ cells/insert) sublines for 24 h and 48 h, respectively, determined by the area of gap closure (H2023, *p* < 0.001; H522, *p* = 0.005). Scale bar, 200 μm. (**G**) The invasion of the H2023 (4 × 10^4^ cells/well) and H522 (5 × 10^4^ cells/well) sublines over 18 h and 48 h, respectively, assessed through the Transwell assay (H2023, *p* < 0.001; H522, *p* = 0.004). Invasive cells were stained using crystal violet, and 10 random fields of view were quantified at 100× magnification per well. Scale bar, 200 μm. (H) Representative phase-contrast images of the H2023 and H522 sublines. Scale bar, 200 μm. The data in (**A**–**G**) were obtained from n ≥ 3 independent experiments. Statistical differences were calculated through a one-way ANOVA followed by Tukey’s multiple comparison test (* *p* < 0.05, ** *p* < 0.01, *** *p* < 0.001). For clarity, the *p*-values reported in the text only compare wild-type and mutant groups. The original Western blot figures can be found in Supplementary Materials.

To evaluate the potential oncogenic activity of *ARAF* p.S214C in lung adenocarcinoma cell lines, the *EGFP* control vector, the *ARAF* wild-type (WT), and *ARAF* p.S214C were ectopically expressed in H2023 and H522 cells. The Western blot analysis showed that the *ARAF* p.S214C mutation elevated the MEK and ERK phosphorylation compared to that in the *EGFP* and *ARAF*-WT controls, indicating activation of the RAF-MEK-ERK pathway (Figure 2A). The *ARAF* p.S214C cells also demonstrated a higher proliferation rate (Figure 2B) and a shorter doubling time than those in the control groups (Figure 2C), suggesting that *ARAF* p.S214C could be an oncogenic driver of cell growth. The *ARAF* p.S214C cells also showed an enhanced colony formation ability compared to that in the controls (Figure 2D). In the 3D spheroid cultures, the *ARAF* p.S214C mutant-derived spheroids exhibited an increased spheroid area and cell viability (luminescence intensity) over a 10-day growth period (Figure 2E). The *ARAF* p.S214C mutant cells also showed increased migratory (Figure 2F) and invasive abilities (Figure 2G), which may be justified by their morphological characteristics of being more elongated and spindle-shaped (Figure 2H), suggesting loose cell–cell interactions that may contribute to enhanced motility and invasiveness. These findings underscored the enhanced oncogenic potential and aggressiveness associated with the *ARAF* p.S214C mutation.

### 3.3. ARAF p.S214C Is Sensitive to Sorafenib In Vitro

**Figure 3:**
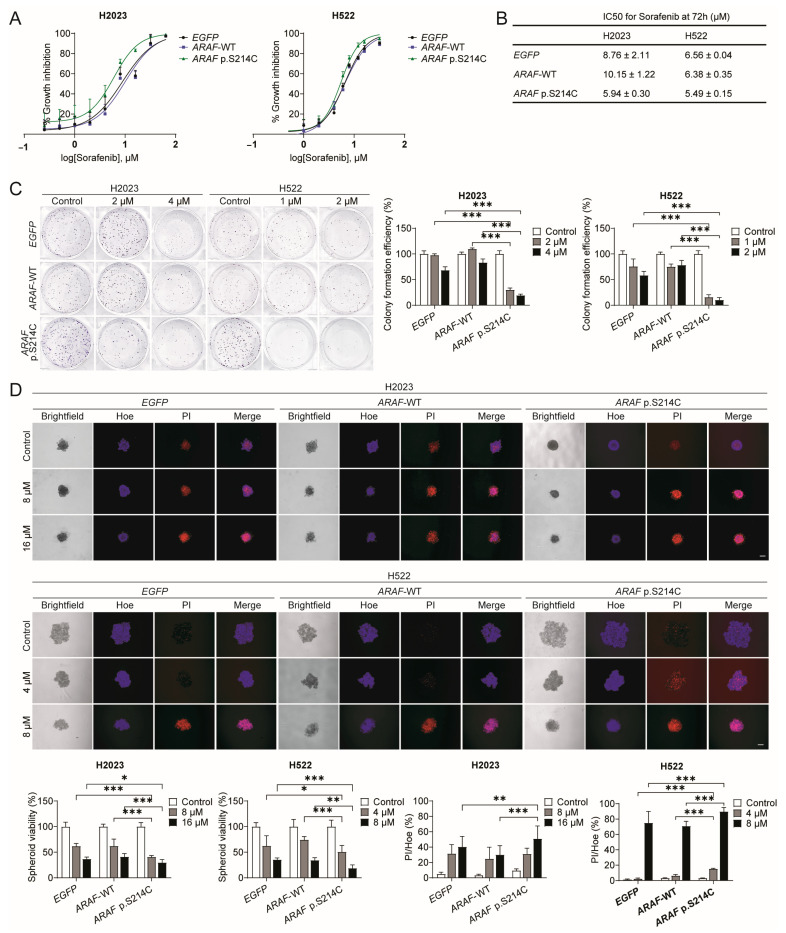
*ARAF* p.S214C mutant cells show sensitivity to sorafenib in vitro. (**A**) Dose–response curves and (**B**) half-maximal inhibitory concentrations (IC_50_) for sorafenib at 72 h in H2023 (3000 cells/well) and H522 cells (6000 cells/well) ectopically expressing *EGFP*, *ARAF*-WT, and *ARAF* p.S214C, denoted as sublines below (IC_50_ values: H2023, *p* = 0.0237; H522, *p* = 0.0064). (**C**) The colony formation efficiency of H2023 (1000 cells/well, 7 days) and H522 (1000 cells/well, 14 days) sublines following treatment with DMSO control or sorafenib at different doses (H2023: *p* < 0.001; H522: *p* < 0.001). (**D**) Spheroid formation efficiency of H2023 and H522 (both 1000 cells/well, 4 days of spheroid growth) sublines following treatment with DMSO control or sorafenib for 72 h. Spheroids were stained with Hoechst 33342 and propidium iodide. The apoptotic subpopulation in the spheroids is presented as the ratio of PI^+^ cells, representing apoptotic cells, to Hoe^+^ cells, representing the total cell count (H2023: *p* < 0.001; H522: *p* < 0.001). Spheroid viability was determined according to the luminescence signal in the CellTiter-Glo 3D Cell Viability assay (H2023: *p* < 0.001; H522: *p* = 0.001). Scale bar, 200 μm. The data in (**A**–**D**) were obtained from n ≥ 3 independent experiments. The statistical difference was calculated through a one-way ANOVA followed by Tukey’s multiple comparison test (* *p* < 0.05, ** *p* < 0.01, *** *p* < 0.001). For clarity, the *p*-values reported in the text only compare wild-type and mutant groups.

Subsequently, the responses of *ARAF* p.S214C to sorafenib treatment were evaluated in vitro. The *ARAF* p.S214C cells demonstrated heightened sensitivity to sorafenib, as shown by the dose–response curves (Figure 3A), with the significantly reduced IC_50_ value reflecting increased susceptibility to sorafenib-induced growth inhibition compared to that in the *EGFP* and *ARAF*-WT controls (Figure 3B). Similarly, the *ARAF* p.S214C cells were more sensitive to the MEK inhibitor PD98059 (Figure S1), in which MEK is a downstream effector of RAF, suggesting that the sorafenib sensitivity may depend on the kinase activity of ARAF. Moreover, the *ARAF* p.S214C mutant cells exhibited a significantly diminished ability to form colonies following the sorafenib treatment compared to that in the control groups (Figure 3C), highlighting the pronounced inhibitory effect of sorafenib on the proliferative capacity of the mutant cells. In addition, a marked increase in the PI^+^ apoptotic subpopulation and a reduction in viable cells in the *ARAF* p.S214C spheroids were observed upon sorafenib treatment (Figure 3D), indicating elevated apoptosis and compromised cell viability in the 3D spheroid cultures. Together, these results demonstrated that *ARAF* p.S214C mutant cells exhibit enhanced sensitivity to sorafenib in terms of their cell viability and colony and spheroid formation capabilities following sorafenib treatment compared to those in control and wild-type counterparts.

### 3.4. ARAF p.S214C Is Sensitive to Sorafenib In Vivo

**Figure 4:**
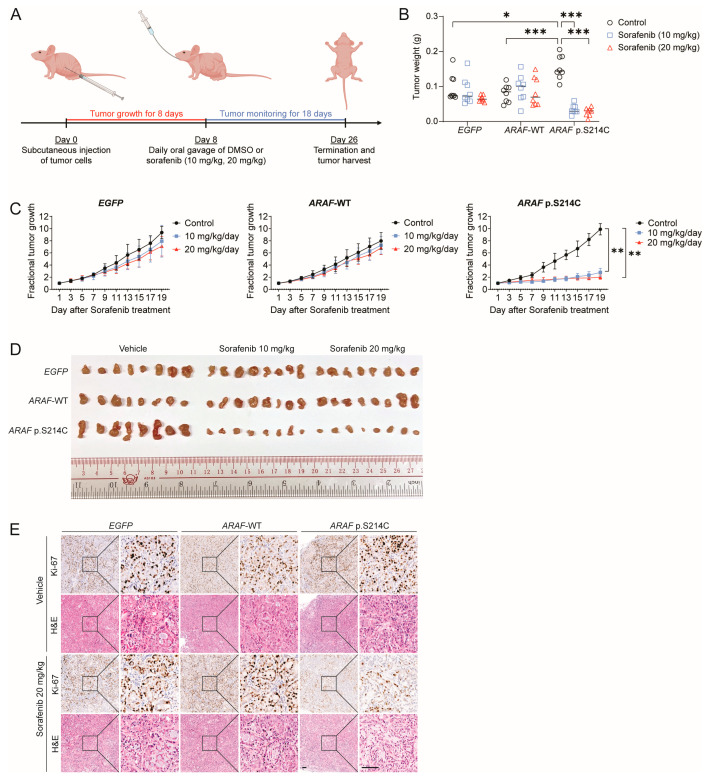
*ARAF* p.S214C mutant cells show sensitivity to sorafenib in vivo. (**A**) The dosing schedule for sorafenib treatment of the mice. H2023 cells expressing *EGFP*, *ARAF*-WT, and *ARAF* p.S214C were subcutaneously inoculated into 4- to 6-week-old male Nu/J mice, respectively (2 × 10^6^ cells per inoculation). Mice with the respective xenografts were treated with sorafenib (10 mg/kg or 20 mg/kg) or cremophor EL/95% ethanol (as the vehicle) daily via oral gavage for 18 days. Each group comprised 4 mice, with each bearing 2 tumors (n = 8 tumors per group). (**B**) Column scatter plots showing the individual tumor volumes at the end of the experiment (p.S214C control vs. WT control, *p* < 0.001; p.S214C control vs. p.S214C sorafenib, *p* < 0.001). Horizontal lines indicate the mean tumor weight. (**C**) The fractional tumor growth curves for the mice xenografts expressing *EGFP*, *ARAF*-WT, and *ARAF* p.S214C upon sorafenib or DMSO vehicle treatment for 18 days (20 mg/kg sorafenib, p.S214C vs. WT, *p* = 0.003). (**D**) Images of the tumors at the experimental endpoint. (**E**) Representative images of immunohistochemistry staining of the proliferation marker Ki-67 and H&E staining of the xenografts upon the sorafenib (20 mg/kg) or vehicle treatment. 100× and 400×; scale bar, 100 μm. The statistical difference was calculated through a one-way ANOVA followed by Tukey’s multiple comparison test (* *p* < 0.05, ** *p* < 0.01, *** *p* < 0.001). For clarity, the *p*-values reported in the text only compare the wild-type and mutant groups.

Tumor xenografts from H2023 cells expressing *EGFP*, *ARAF*-WT, and *ARAF* p.S214C in immunodeficient Nu/J mice were also generated, and their in vivo tumorigenicity and sorafenib response were compared. The tumor-bearing mice were treated with sorafenib at 10 mg/kg and 20 mg/kg or the vehicle daily via oral administration for 18 days (Figure 4A). The final tumor weights and tumor growth rates of the *ARAF* p.S214C xenografts were significantly higher compared to those of the *EGFP* and *ARAF*-WT xenografts in the vehicle groups, indicating that the *ARAF* p.S214C xenografts exhibited enhanced oncogenic growth in vivo (Figure 4B,C). Upon sorafenib treatment, the tumor growth in the mice bearing the *ARAF* p.S214C xenografts was markedly suppressed, as demonstrated by a reduced tumor weight and fractional tumor growth, whereas no significant changes in tumor growth were observed for the *EGFP* and *ARAF*-WT xenografts (Figure 4B,C). It was evident that the tumor sizes of the *ARAF* p.S214C xenografts were significantly larger in the absence of the drug treatment but showed considerable shrinkage following the sorafenib treatment at both doses (Figure 4D). Sorafenib did not cause substantial toxicity in terms of body weight (Figure S2). Subsequent IHC and H&E analyses confirmed the ARAF expression in the *ARAF*-WT and *ARAF* p.S214C xenografts (Figure S3). Significantly reduced cell proliferation (Ki-67) was observed in the sorafenib-treated *ARAF* p.S214C xenografts compared to that in the vehicle group (Figure 4E). Consistent with the in vitro findings, our in vivo data provided evidence that *ARAF* p.S214C is an oncogenic driver and shows sorafenib sensitivity.

### 3.5. Transcriptomic and Proteomic Analyses of the ARAF-WT and ARAF p.S214C Cells in the Absence or Presence of Sorafenib Treatment

To understand the molecular mechanisms underlying the oncogenic role and sorafenib sensitivity of *ARAF* p.S214C, the gene and protein expression profiles of the *ARAF*-WT- and *ARAF* p.S214C-expressing H2023 cells, treated with sorafenib or DMSO, were investigated using RNA-Seq and the RPPA (Figure S4A,B), as denoted by MUcon (*ARAF* p.S214C + DMSO), MUsor (*ARAF* p.S214C + sorafenib), WTcon (*ARAF*-WT + DMSO), and WTsor (*ARAF*-WT + sorafenib).

**Figure 5:**
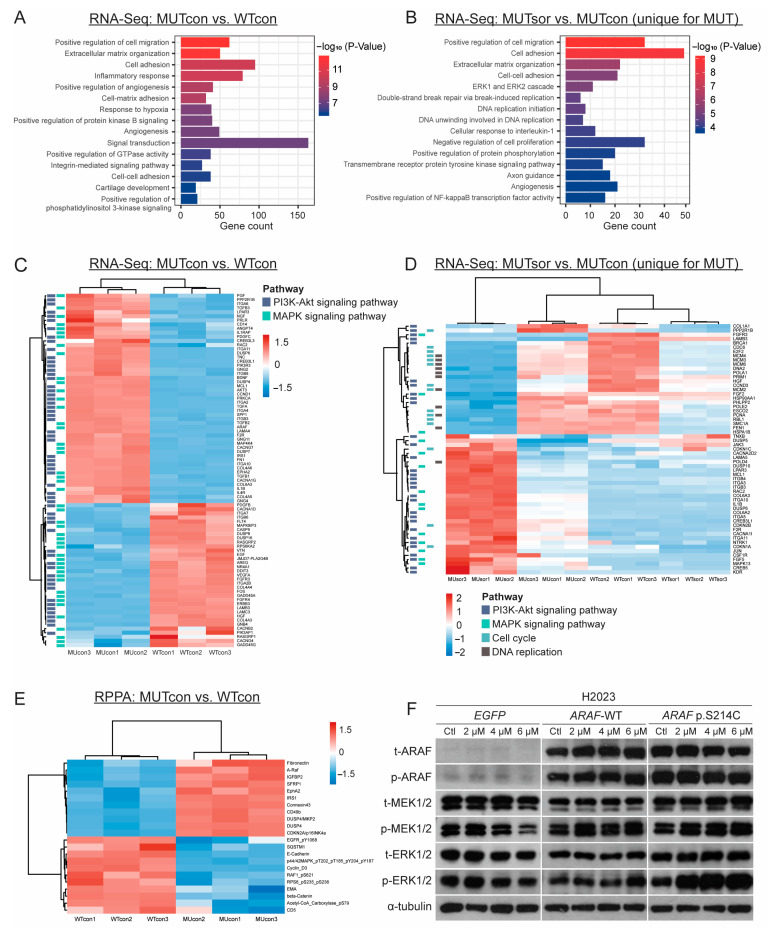
Transcriptomic and proteomic analyses of *ARAF*-WT and *ARAF* p.S214C mutant cells with or without sorafenib treatment. RNA-Seq and RPPA were performed for H2023 cells expressing *ARAF*-WT and *ARAF* p.S214C treated with vehicle control or 4 µM of sorafenib for 72 h. Three replicates were used per group. (**A**) Bar plot of top 15 enriched GO BP terms for DEGs between *ARAF*-WT and *ARAF* p.S214C cells, ranked in order of significance based on *p*-values. (**B**) Heatmap displaying DEGs within selected pathways of interest between *ARAF*-WT and *ARAF* p.S214C cells without sorafenib treatment. (**C**) Bar plot of top 15 enriched GO BP terms for unique DEGs between *ARAF*-WT and *ARAF* p.S214C cells with and without sorafenib treatment, ranked in order of significance based on *p*-values. (**D**) Heatmap of *ARAF* p.S214C-mutant-specific DEGs in specified enriched pathways in response to sorafenib treatment. (**E**) Heatmap presenting the differential protein expression patterns identified from the RPPA analysis between the *ARAF*-WT and *ARAF* p.S214C cells without sorafenib treatment. (**F**) The Western blot analysis showing the expression levels of t-ARAF, p-ARAF, t-MEK1/2, p-MEK1/2, t-ERK1/2, and p-ERK1/2 in H2023 cells ectopically expressing *EGFP*, *ARAF*-WT, and *ARAF* p.S214C upon sorafenib treatment. α-tubulin was included as a loading control. The original Western blot figures can be found in Supplementary Materials.

The oncogenic impact of the *ARAF* p.S214C mutation on the gene expression profiles was examined by identifying the differentially expressed genes (DEGs) between the MUcon and WTcon groups. From the RNA-Seq data, 887 upregulated genes (Figure S5B), including *ARAF*, and 1376 downregulated genes were identified in MUcon compared to WTcon. The GO enrichment analysis highlighted significant alterations in genes related to tumor invasion and metastasis, including cell migration, adhesion, and angiogenesis (Figure 5A, Tables S3 and S4). The KEGG analysis also revealed that the *ARAF* p.S214C-mutation-deregulated genes were enriched in oncogenic signaling pathways, including the MAPK and phosphatidylinositol 3-kinase (PI3K)-Akt signaling pathways (Figure 5C, Table S5). From the RPPA analysis, 11 differentially upregulated and 11 downregulated proteins were identified in the *ARAF* p.S214C cells (Figure 5E and Figure S5C, Table S12). Consistent with the RNA-Seq data, ARAF and proteins associated with cell adhesion and migration were upregulated in the *ARAF* p.S214C cells, including connexin 43, EPH receptor A2, and fibronectin. Proteins associated with cell adhesion were also downregulated, including epithelial membrane antigen (EMA), E-cadherin, and β-catenin, suggesting the disruption of cell integrity and increased migratory potential in the *ARAF* p.S214C cells. These results demonstrated significant alterations in cell adhesion, migration, and oncogenic signaling pathways, suggesting that the *ARAF* p.S214C mutation may confer enhanced tumorigenic potential and aggressiveness, which correlates with our in vitro and in vivo findings.

To explore the molecular basis of sorafenib sensitivity, the DEGs between the MUsor and MUcon groups and the WTsor and WTcon groups were examined. In the MUsor group, 676 and 246 DEGs were upregulated or downregulated, respectively, compared to MUcon, which were unique to the MUsor vs. MUcon comparison but not WTsor vs. WTcon (Figure S5A). Both the GO analysis (Figure 5B, Table S6) and the KEGG analysis (Figure 5C, Table S7) showed that the uniquely downregulated genes were enriched in DNA replication and cell cycle processes. Furthermore, 13 genes with contrasting expression were identified in the sorafenib-treated *ARAF* p.S214C and *ARAF*-WT groups (Table S8). Notably, CDKN1A, encoding p21, a pivotal negative regulator of the cell cycle, was significantly upregulated in MUsor vs. MUcon but downregulated in WTsor vs. WTcon in both the RNA-Seq and RPPA data, suggesting inhibition of the cell cycle through negative regulation by p21 in MUsor (Tables S8 and S12, Figure S6). It was also observed that 6 out of 10 genes in the Cdc45-MCM-GINS (CMG) helicase family were uniquely downregulated in MUTsor, including MCM2, MCM3, MCM4, MCM6, GINS1, and GINS2 (Table S9), which are essential for DNA replication. In addition, enrichment in the signaling pathways of the ERK1/2 cascade and PI3K-Akt signaling pathways was also identified (Figure 5D). Paradoxically, the Western blot analysis showed that while sorafenib downregulated phosphorylated MEK and ERK proteins in the *EGFP* cells, upregulation of these proteins was observed in the sorafenib-treated *ARAF*-WT and *ARAF* p.S214C cells (Figure 5F). This may suggest a feedback mechanism that activates MEK/ERK signaling with non-canonical inhibitory effects or the dominance of alternative pathways such as those involving p21 and CMG helicases that counteract the canonical proliferative signals of MEK/ERK signaling in ARAF-expressing cells. Overall, these findings suggest that the increased sorafenib sensitivity observed in the *ARAF* p.S214C cells could be attributed to disruption of the cell cycle and DNA replication processes, while deregulation of the ERK1/2 and PI3K-Akt signaling pathways was observed in the *ARAF* p.S214C mutants regardless of sorafenib treatment.

## 4. Discussion

In recent years, extensive genomic characterization has revealed various actionable mutations in lung cancer, including epidermal growth factor receptor (EGFR), Kirsten rat sarcoma virus (KRAS), and anaplastic lymphoma kinase (ALK) mutations [15]. However, despite advancements in the development of targeted therapies, lung cancer remains a deadly disease due to its complex genetic landscape and heterogeneous responses to treatments [16,17]. Currently, the only precision medicine options for RAF-mutated lung cancer are dabrafenib (combined with trametinib) [18] and encorafenib (combined with binimetinib) [19], both of which target the *BRAF* p.V600 mutation in combination with MEK inhibitors. The availability of ARAF-targeted therapies remains limited. Previously, a case report revealed an exceptional sorafenib response with near-complete tumor regression in a lung adenocarcinoma patient with an *ARAF* p.S214C mutation [14], suggesting the potential therapeutic value of this well-characterized multi-kinase inhibitor for lung cancer patients with *ARAF* mutations.

In this study, the oncogenic potential and sorafenib sensitivity of the *ARAF* p.S214C mutation were elucidated in lung cancer models. The *ARAF* p.S214C mutation was found to activate the Raf-MEK-ERK signaling pathway and demonstrated enhanced oncogenic potential in terms of cell proliferation, colony and spheroid formation efficiency, and migration and invasion capabilities, which aligns with previous findings that underscore the critical role of this pathway in tumorigenesis [5]. Importantly, functional analyses revealed that this mutation exhibited heightened sensitivity to sorafenib treatment both in vitro and in vivo, which is concordant with the report of the clinically exceptional responder with the *ARAF* p.S214C mutation [14].

Transcriptomic and proteomic analyses of the *ARAF* p.S214C and *ARAF*-WT cells also corroborated our functional data, revealing alterations in the genes or proteins involved in oncogenesis in the *ARAF* p.S214C cells. Upregulation of molecules that regulate cell adhesion and migration—connexin 43, EPH receptor A2, and fibronectin—as well as downregulation of molecules that reduce cell–cell adhesion—EMA, E-cadherin, and β-catenin—were observed in the *ARAF* p.S214C cells compared to the *ARAF*-WT cells. These changes potentially drive the transition from an epithelial to a mesenchymal phenotype, leading to enhanced invasiveness and tumor aggressiveness [20,21,22,23,24,25]. Moreover, the identification of CDKN1A and CMG helicases as unique regulators in the sorafenib-treated *ARAF* p.S214C cells, but not the *ARAF*-WT cells, suggested that the sorafenib sensitivity conferred by the *ARAF* p.S214C mutation was associated with the inhibition of cell cycle and DNA replication processes. CDKN1A encodes for p21, a negative cell cycle regulator within the cyclin-dependent kinase inhibitor (CKI) family, which interacts with cyclin-dependent kinases (CDKs) and proliferating cell nuclear antigen (PCNA) [26]. The CMG complex is an eukaryotic replication helicase responsible for initiating DNA replication [27]. Upregulation of these molecules has been shown to trigger cell cycle arrest due to the accumulation of unrepaired DNA lesions [28,29], possibly explaining the heightened sorafenib sensitivity of *ARAF* p.S214C cells.

Meanwhile, deregulation of the MEK-ERK and PI3K-Akt signaling pathways was observed in the *ARAF* p.S214C mutants regardless of sorafenib treatment. The activation of MEK-ERK signaling in the untreated *ARAF* p.S214C mutants was consistent with our in vitro findings, suggesting that enhanced tumor aggressiveness might be associated with increased MEK-ERK signaling. However, paradoxical upregulation of phosphorylated MEK and ERK proteins was observed in the sorafenib-treated *ARAF*-WT and *ARAF* p.S214C cells but not in the sorafenib-treated *EGFP* cells. This may implicate potential feedback that triggers the non-canonical antiproliferative and pro-apoptotic effects of activated MEK-ERK signaling in ARAF-expressing cells [30,31]. Another possible explanation is that sorafenib may exert cytostatic and cytotoxic effects in ARAF-expressing cells via alternative pathways that are more dominant, such as those involving p21 and CMG helicases, as highlighted by the RNA-Seq and RPPA findings. These pathways promote cell cycle arrest and potentially override the canonical proliferative and survival signals typically driven by MEK-ERK activation. The exact mechanism warrants more in-depth investigation of the downstream molecular effectors and interplay between these signaling pathways.

Although *ARAF* mutations only constitute ~1.4% of lung cancers, with the *ARAF* p.S214C mutation occurring in ~0.03% of lung adenocarcinoma cases [8,32], our findings revealed the therapeutic potential of sorafenib in a distinct subgroup of patients. This expands the repertoire of precision medicine therapy in lung cancer and demonstrates that even rare mutations could have profound implications for cancer aggressiveness and treatment responses [8,33,34]. Given that lung cancer is the most frequent type of cancer worldwide, with ~2.5 million new cases in 2022 [1], an estimated 750 lung cancer patients per year may harbor the *ARAF* p.S214C mutation and could potentially benefit from sorafenib treatment.

Importantly, this is the first study to systematically investigate the oncogenicity and sorafenib response of the *ARAF* p.S214C mutation in lung cancer. Apart from the functional assays that demonstrate its oncogenic role and sorafenib sensitivity, this study also provides novel perspectives into the molecular mechanism underlying these effects at the transcriptomic and proteomic levels. Moreover, these findings resonate with previous non-precision-based clinical trials that have led to accelerated approval of precision medications that are off-label but widely prescribed in other cancer types, such as gefitinib for EGFR-mutated non-small-cell lung cancer (NSCLC) [35] and crizotinib for ALK-rearranged NSCLC [36]. Since sorafenib is already an FDA-approved therapy for hepatocellular carcinoma, renal cell carcinoma, and thyroid cancer [10,11,12] with a well-characterized safety profile, it could readily be repurposed as a targeted therapy in the specific lung cancer subgroup harboring the *ARAF* p.S214C mutation without the need for the extensive safety trials typically required for novel drugs. While traditional drug development typically takes 10–15 years to reach FDA approval [37], investigating exceptional responder (ER) cases from non-precision-based clinical trials with scientific characterization and pharmacologic credentialing in cancer models may generate compelling evidence of gene–drug sensitivity [38,39]. This insight may inform clinical trial design and potentially lead to the clinical application of precision treatments in as little as 2–6 years.

However, several limitations should be acknowledged when interpreting the results. First, the experimental data from this work was primarily obtained through in vitro and in vivo studies using two lung adenocarcinoma cell lines and corresponding xenograft models. To establish the clinical relevance of the *ARAF* p.S214C mutation, additional clinicopathological and molecular studies using patient-derived xenograft models and clinical specimens are essential. Second, the molecular mechanisms underlying the oncogenicity and sorafenib sensitivity of the *ARAF* p.S214C mutation remain incompletely understood. Although transcriptomic and proteomic analyses were performed, further validation using RT-qPCR, Western blot, or other assays is necessary to delineate the specific downstream signaling pathways. Third, sorafenib was the primary therapeutic agent evaluated in this study. While sorafenib monotherapy showed efficacy against the *ARAF* p.S214C mutant cells, future research could explore other multi-kinase inhibitors and combination strategies [40,41,42], which may enhance treatment outcomes further. Fourth, this study only characterized the *ARAF* p.S214C mutation in the context of lung cancer. It remains to be determined whether this mutation or other *ARAF* p.S214 mutations exhibit similar sorafenib sensitivity across different cancer types [34,43]. Broadening the scope of investigation could uncover additional *RAF* mutation targets and tumor types for which agents like sorafenib could be effective.

## 5. Conclusions

In conclusion, our study elucidates the oncogenic role of the *ARAF* p.S214C mutation in lung cancer and suggests the potential therapeutic avenue of its relationship with sorafenib, reinforcing the necessity of continuous research to refine patient stratification based on rare mutations. Notably, this study may provide strong scientific evidence to establish the *ARAF* p.S214C mutation as a novel biomarker for predicting sorafenib’s efficacy in lung cancer and inform future clinical trials aiming to evaluate the sorafenib responses in *ARAF*-mutated lung cancer patients. The identification of the *ARAF* p.S214C mutation as a potential biomarker for sorafenib responsiveness through the analysis of an exceptional responder case underscores the importance of comprehensive genetic profiling to guide and accelerate personalized cancer treatment strategies.

## Data Availability

The data from the American Association for Cancer Research (AACR) Project Genomics Evidence Neoplasia Information Exchange (GENIE) public database cohort v14.1 (https://www.aacr.org/professionals/research/aacr-project-genie/aacr-project-genie-data/ (accessed on 5 November 2024)) are publicly available. The data generated in this study are available within the article and its Supplementary Materials. Original data generated in this study can be made available on request by the corresponding author.

## Supplementary Materials

The following supporting information can be downloaded at https://www.mdpi.com/article/10.3390/cancers17132246/s1, Western blot_Raw Data; STR_NCI-H2023; STR_NCI-H522; STR_PLAT-A; Figure S1: *ARAF* p.S214C mutant cells show sensitivity to PD98059 in vitro; Figure S2: Body weight of mice xenografts expressing *EGFP*, *ARAF*-WT, and *ARAF* p.S214C upon sorafenib or vehicle treatment for 18 days; Figure S3: Representative images of immunohistochemistry staining of total (t)-ARAF and phospho (p)-ARAF in mice xenografts expressing *EGFP*, *ARAF*-WT, and *ARAF* p.S214C; Figure S4: PCA of RNA-Seq and RPPA data; Figure S5: Comparative analysis of RNA-Seq and RPPA data; Figure S6: Relative protein expression levels of p21 among H2023 cells expressing *EGFP*, *ARAF*-WT, and *ARAF* p.S214C under both sorafenib-treated and untreated conditions as determined through RPPA; Table S1: A list of the resources used in this study; Table S2: Summary of p-values from pairwise statistical comparisons among mutant, wild-type and EGFP control vector groups; Table S3: GO enrichment analysis of DEGs identified in *ARAF* p.S214C mutant cells compared to *ARAF*-WT cells (RNA-Seq); Table S4: GO enrichment analysis of upregulated DEGs identified in *ARAF* p.S214C mutant cells compared to *ARAF*-WT cells (RNA-Seq); Table S5: KEGG enrichment analysis of DEGs identified in *ARAF* p.S214C mutant cells compared to *ARAF*-WT cells (RNA-Seq); Table S6: GO enrichment analysis of uniquely downregulated DEGs in sorafenib-treated *ARAF* p.S214C mutant cells compared to non-treated cells (RNA-Seq); Table S7: KEGG pathway enrichment analysis of uniquely downregulated DEGs in sorafenib-treated *ARAF* p.S214C mutant cells compared to non-treated cells (RNA-Seq); Table S8: DEGs with contrasting expression patterns in sorafenib-treated *ARAF* p.S214C mutant cells compared to those in non-treated cells (RNA-Seq); Table S9: Unique DEGs in sorafenib-treated *ARAF* p.S214C mutant cells compared to *ARAF*-WT cells (RNA-Seq); Table S10: Protein targets used in RPPA analysis; Table S11: Protein expression profiles in RPPA analysis; Table S12: Differentially expressed proteins identified in *ARAF* p.S214C mutant cells compared to *ARAF*-WT cells with or without sorafenib treatment (RPPA).

## Abbreviations

The following abbreviations are used in this manuscript:

ALK	Anaplastic lymphoma kinase
ANOVA	Analysis of variance
CDK	Cyclin-dependent kinase
CKI	Cyclin-dependent kinase inhibitor
CMG	Cdc45-MCM-GINS
CR	Conserved region
DEG	Differentially expressed gene
ECL	Enhanced chemiluminescence
EGFR	Epidermal growth factor receptor
EMA	Epithelial membrane antigen
ER	Exceptional responder
ERK	Extracellular-signal-regulated kinase
FBS	Fetal bovine serum
H&E	Hematoxylin and eosin
IHC	Immunohistochemistry
KRAS	Kirsten rat sarcoma virus
MEK	Mitogen-activated protein kinase
MTT	3-(4,5-dimethyl-2-thiazolyl)-2,5-diphenyl-2H-tetrazolium bromide
NSCLC	Non-small-cell lung cancer
PCNA	Proliferating cell nuclear antigen
PDGFR	Platelet-derived growth factor receptor
PI3K	Phosphatidylinositol 3-kinase
PLAT-A	Platinum-A
P/S	Penicillin/streptomycin
RBS	RAS-binding domain
RET	Rearranged during transfection
RNA-Seq	RNA sequencing
RPPA	Reverse-phase protein array
VEGFR	Vascular endothelial growth factor receptor
WT	Wild-type
