# Supplementary material for: Oncogenic Activity and Sorafenib Sensitivity of ARAF p.S214C Mutation in Lung Cancer
Source: Cancers (Basel). 2025 Jul 4;17(13):2246. doi: 10.3390/cancers17132246 (PMC12248905; doi:10.3390/cancers17132246)
Supplement: Supplementary file 1 [file cancers-17-02246-s001.zip › Supplementary Figures.pdf]

Oncogenic Activity and Sorafenib Sensitivity of *ARAF* p.S214C Mutation in Lung Cancer

Carol Lee[1], Weixue Mu[1], Xi July Chen[1], Mandy Sze Man Chan[1], Zhishan Chen[1], Sai Fung Yeung[1], Helen Hoi Yin Chan[1], Sin Ting Chow[1], Ben Chi Bun Ko[2], David Wai Chan[3], William Chi Cho[4], Vivian Wai Yan Lui[5,*], Stephen Kwok Wing Tsui[1,*].

[1] School of Biomedical Sciences, The Chinese University of Hong Kong, Hong Kong.

[2] Department of Applied Biology and Chemical Technology, the Hong Kong Polytechnic University, Hong Kong.

[3] School of Medicine, The Chinese University of Hong Kong, Shenzhen.

[4] Department of Clinical Oncology, Queen Elizabeth Hospital, Hong Kong.

[5] Georgia Cancer Center, Department of Medicine, Medical College of Georgia, Augusta University, USA.

* Correspondence: VWYL, wlui@augusta.edu, +706-721-5047; SKWT, kwtsui@cuhk.edu.hk, +852-3943-6381.

Supplementary Figures

A

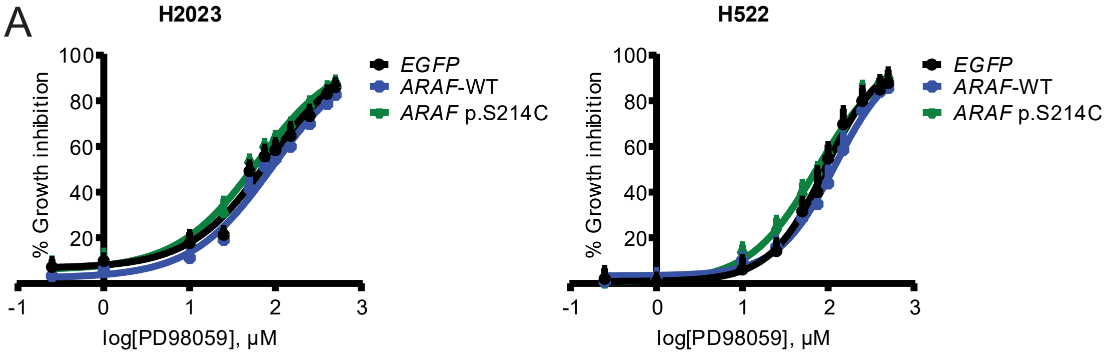

B

	IC50 for PD98059 at 72 h (µM)	
	H2023	H522
EGFP	68.5 ± 12.6	90.3 ± 15.3
ARAF-WT	83.0 ±15.5	116.2 ± 29.5
ARAF p.S214C	52.1 ± 2.72	75.5 ± 6.54

Figure S1. *ARAF* p.S214C mutant cells show sensitivity to PD98059 *in vitro***.** (**A**) Dose-response curves and (**B**) half maximal inhibitory concentrations **(**IC50) for MEK inhibitor PD98059 at 72h in H2023 (3000 cells/well) and H522 cells (6000 cells/well) ectopically expressing *EGFP*, *ARAF*-WT and *ARAF* p.S214C (IC50 values: H2023, p = 0.040; H522, p = 0.096). Data are obtained from n = 3 independent experiments. Statistical difference was calculated by one-way ANOVA followed by Tukey's multiple comparison test.

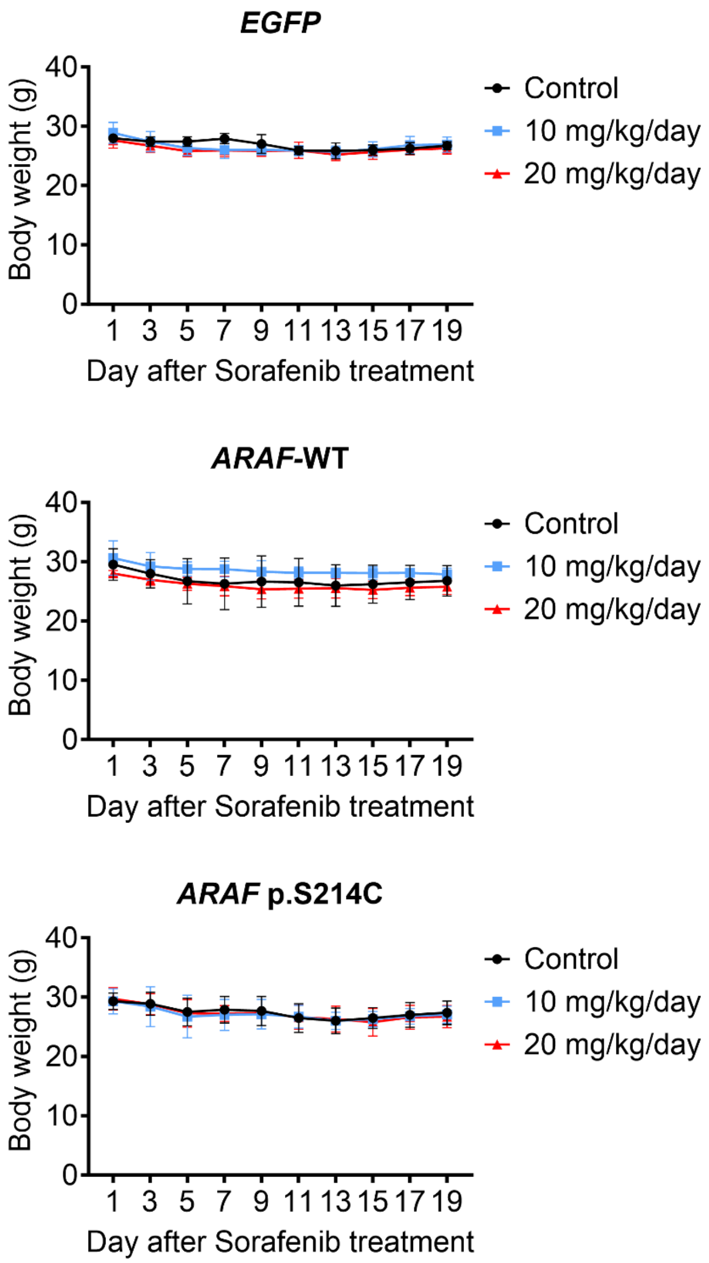

Figure S2. Body weight of mice xenografts expressing *EGFP*, *ARAF*-WT and *ARAF* p.S214C upon sorafenib or vehicle treatments for 18 days**.** Data are shown as mean ± SD (n = 8 tumors per group).

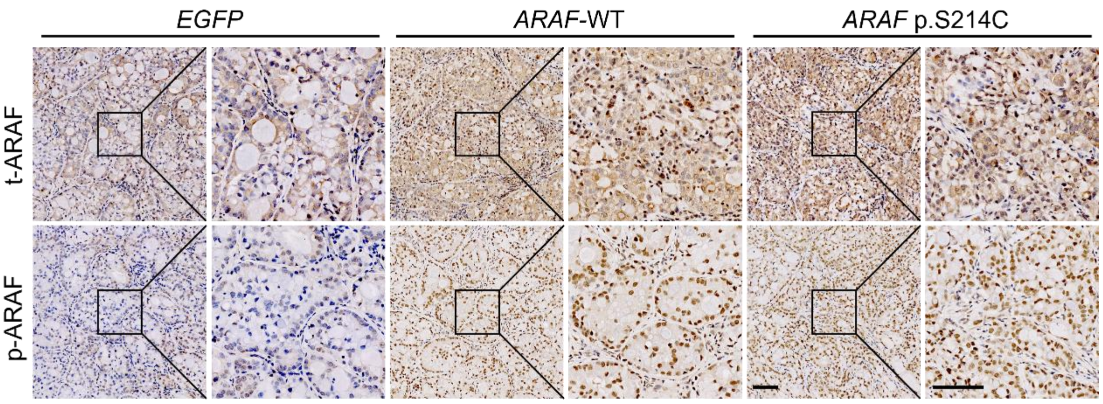

Figure S3. Representative images of immunohistochemistry staining of total (t)-ARAF and phospho (p)-ARAF of mice xenografts expressing *EGFP*, *ARAF*-WT and *ARAF* p.S214C. 200X and 400X, scale bar, 100 μm.

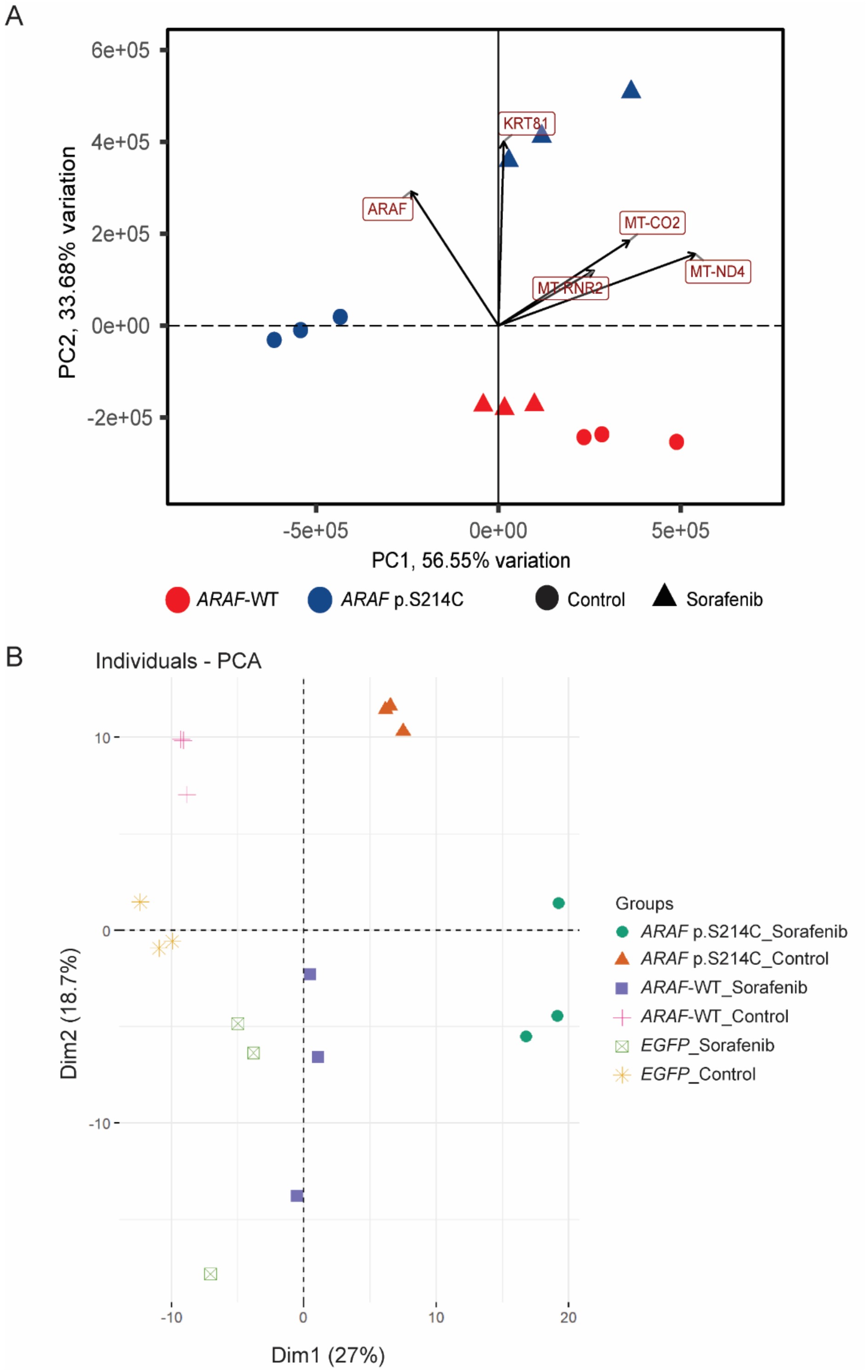

Figure S4. PCA Analysis of RNA-Seq and RPPA data. (**A**) Principal Component Analysis (PCA) plot of RNA-Seq data from samples of *ARAF*-WT and *ARAF* p.S214C mutant cells in the absence or presence of sorafenib. (**B**) PCA analysis of RPPA data from samples of *ARAF*-WT and *ARAF* p.S214C mutant cells, along with *EGFP* control cells, in the absence or presence of sorafenib.

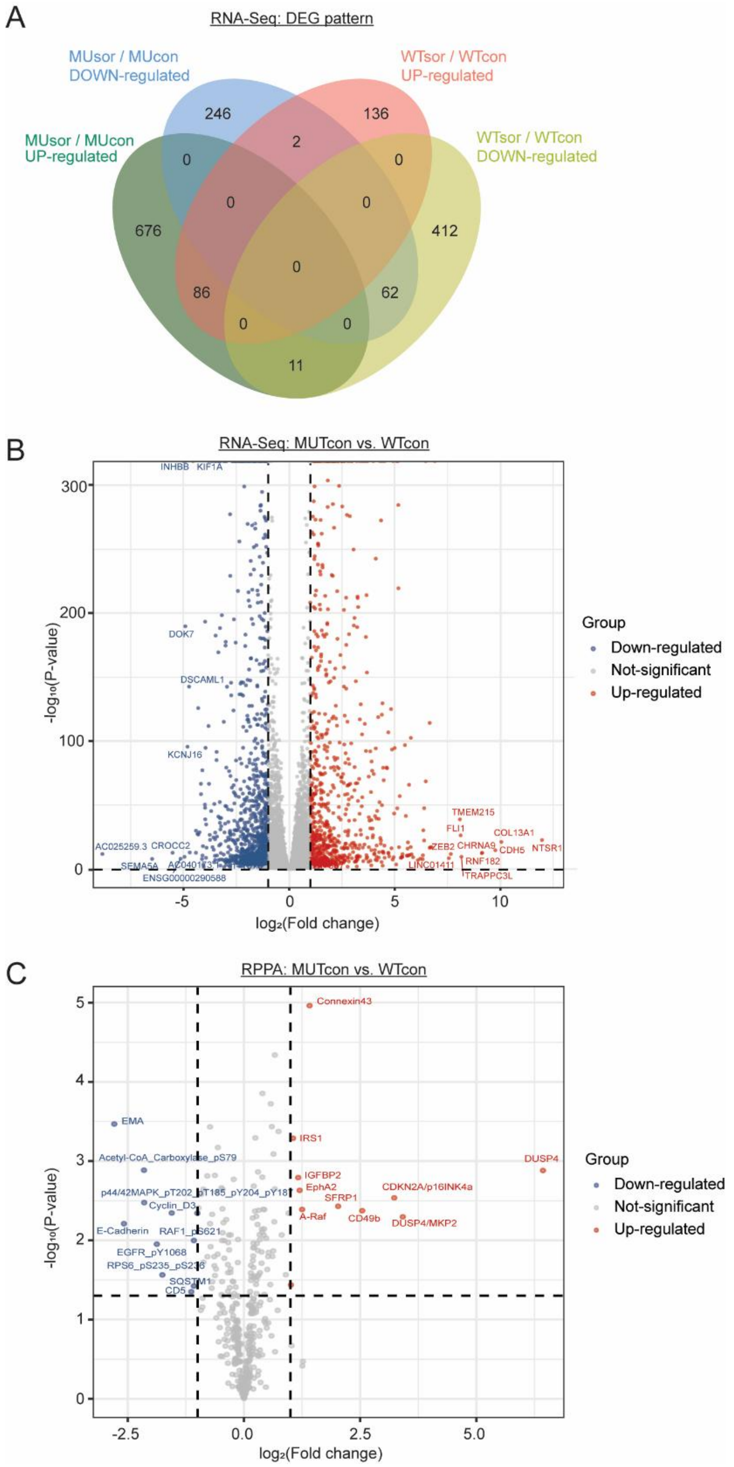

Figure S5. Comparative analysis of RNA-Seq and RPPA data. (**A**) Venn diagram illustrating the intersection analysis of DEGs in MUsor vs. MUcon and WTsor vs. WTcon groups. (**B**) Volcano plot illustrating the DEGs identified from RNA-Seq analysis in *ARAF*-WT and *ARAF* p.S214C cells without sorafenib treatment. (**C**) Volcano plot showcasing the differentially expressed proteins identified from RPPA analysis in *ARAF*-WT and *ARAF* p.S214C cells without sorafenib treatment.

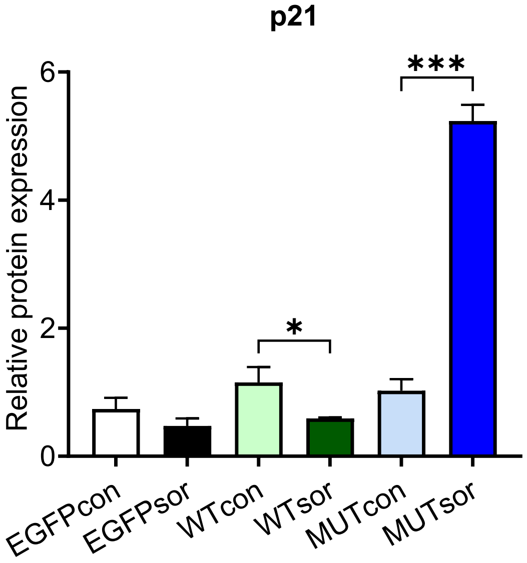

Figure. S6. Relative protein expression levels of p21 among H2023 cells expressing *EGFP*, *ARAF*-WT, and *ARAF* p.S214C under both sorafenib-treated and untreated conditions determined by RPPA. Data are shown as mean ± SD (n = 3 per group).
