# Supplementary figures and images for: Oncogenic Activity and Sorafenib Sensitivity of ARAF p.S214C Mutation in Lung Cancer
Source: Cancers (Basel). 2025 Jul 4;17(13):2246. doi: 10.3390/cancers17132246 (PMC12248905; doi:10.3390/cancers17132246)

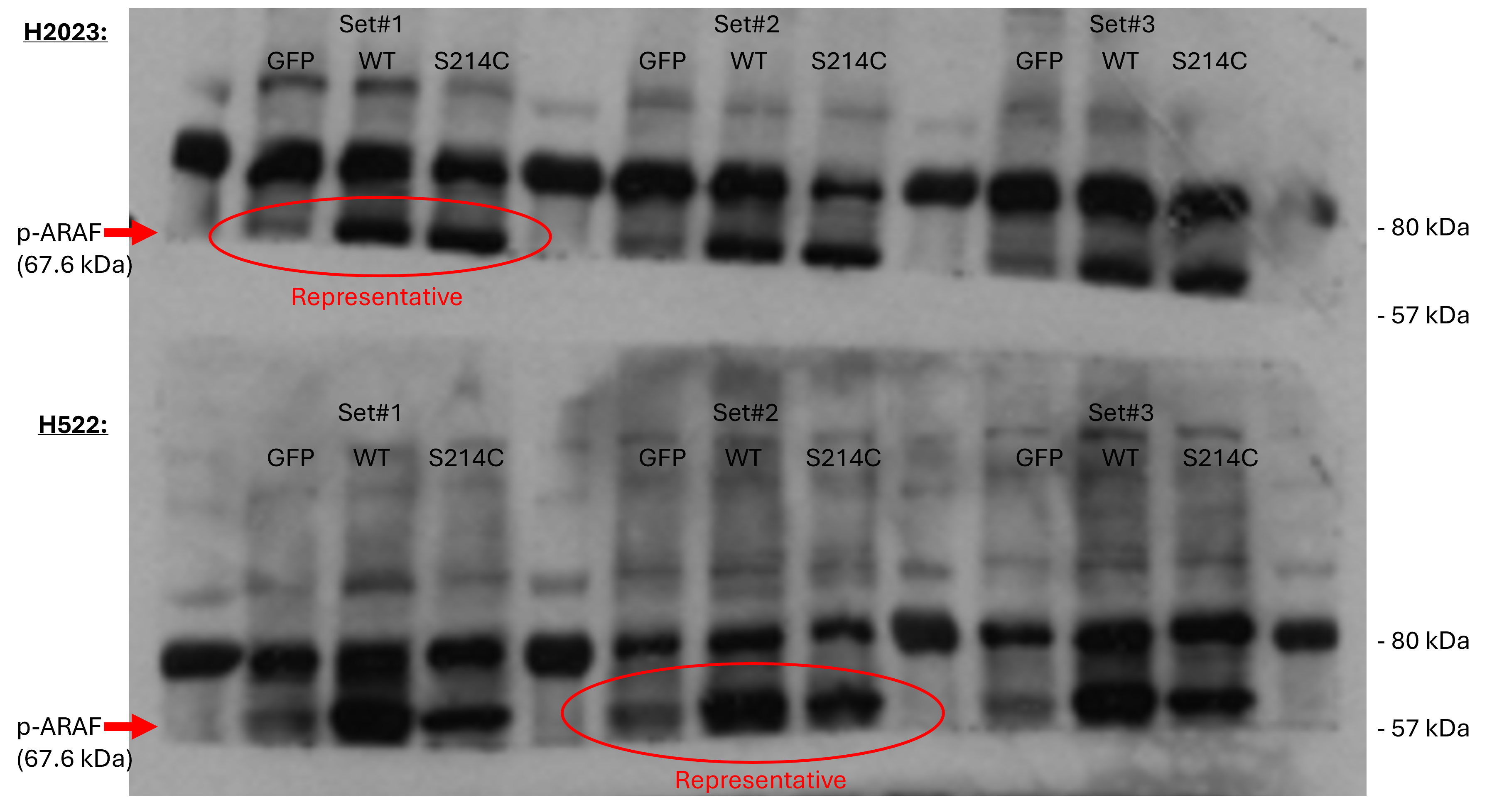

Supplement: Supplementary file 1 [file cancers-17-02246-s001.zip › Western blot_Raw Data/WB_Figure 2A_p-ARAF.tif]

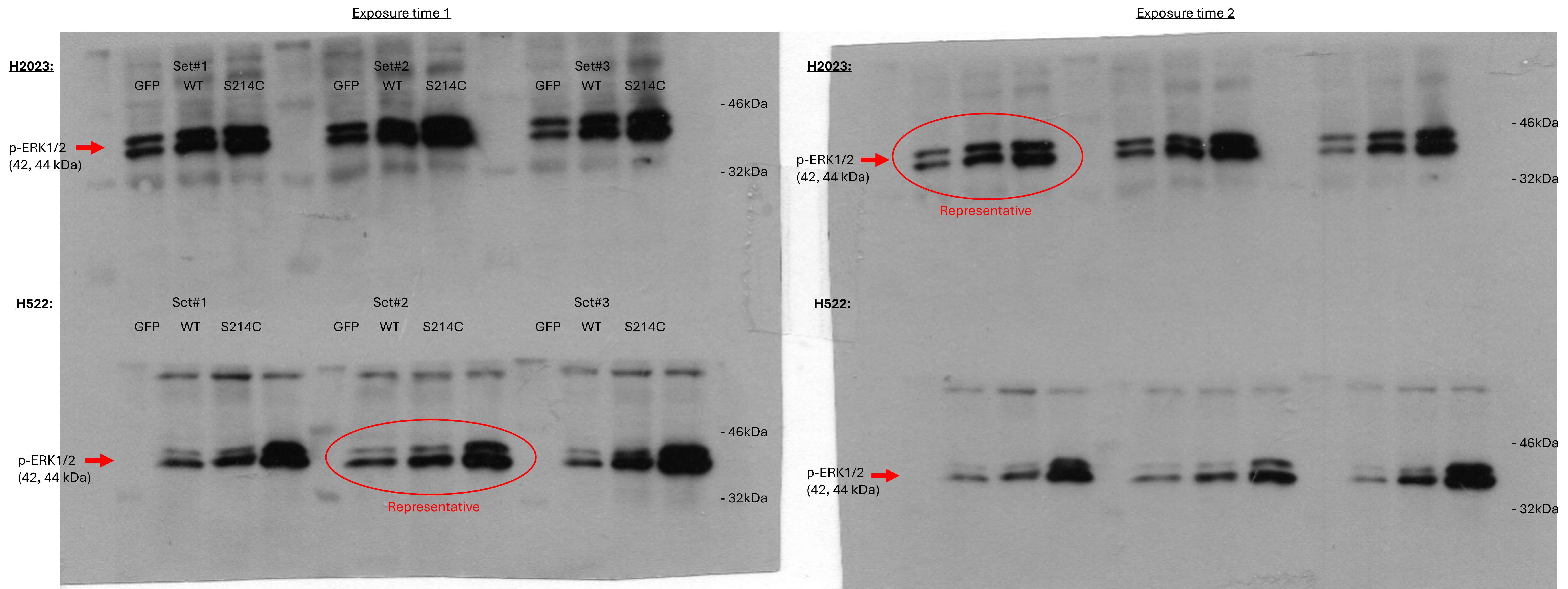

Supplement: Supplementary file 1 [file cancers-17-02246-s001.zip › Western blot_Raw Data/WB_Figure 2A_p-ERK.tif]

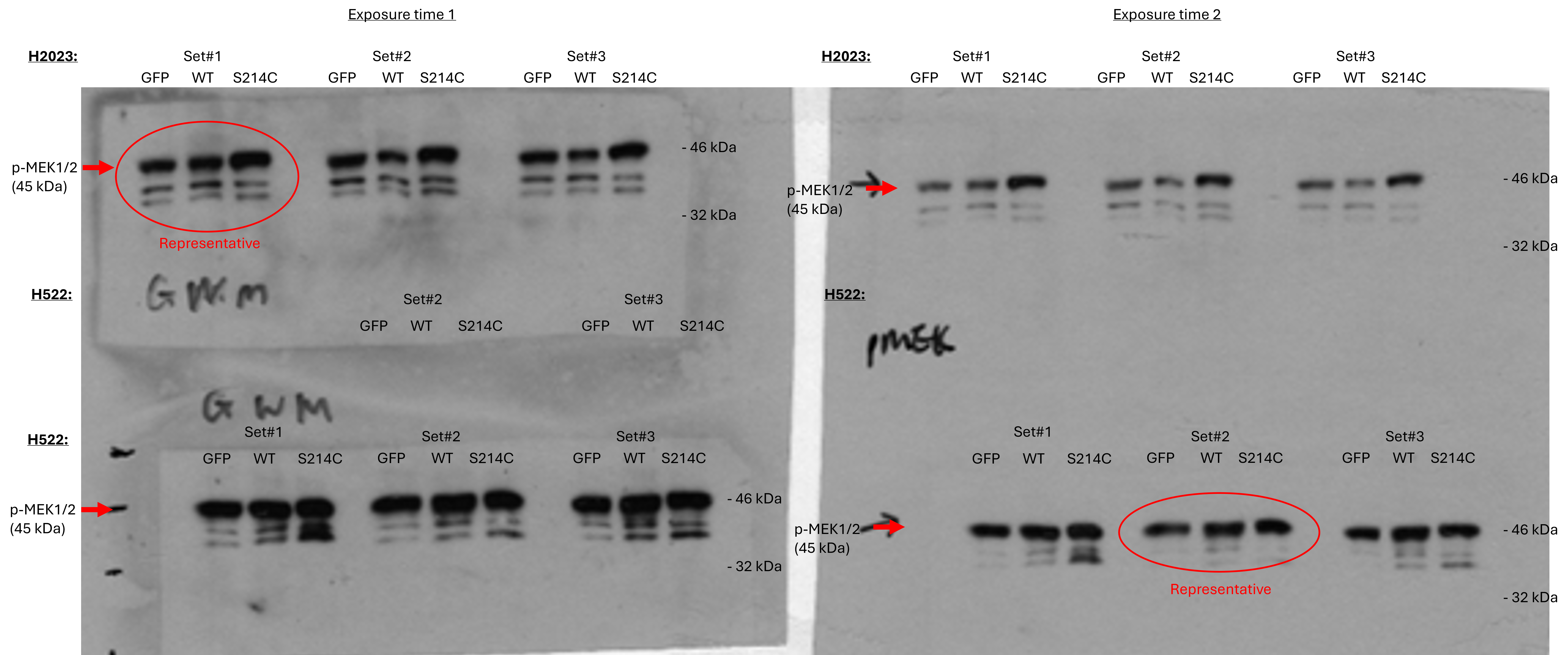

Supplement: Supplementary file 1 [file cancers-17-02246-s001.zip › Western blot_Raw Data/WB_Figure 2A_p-MEK.tif]

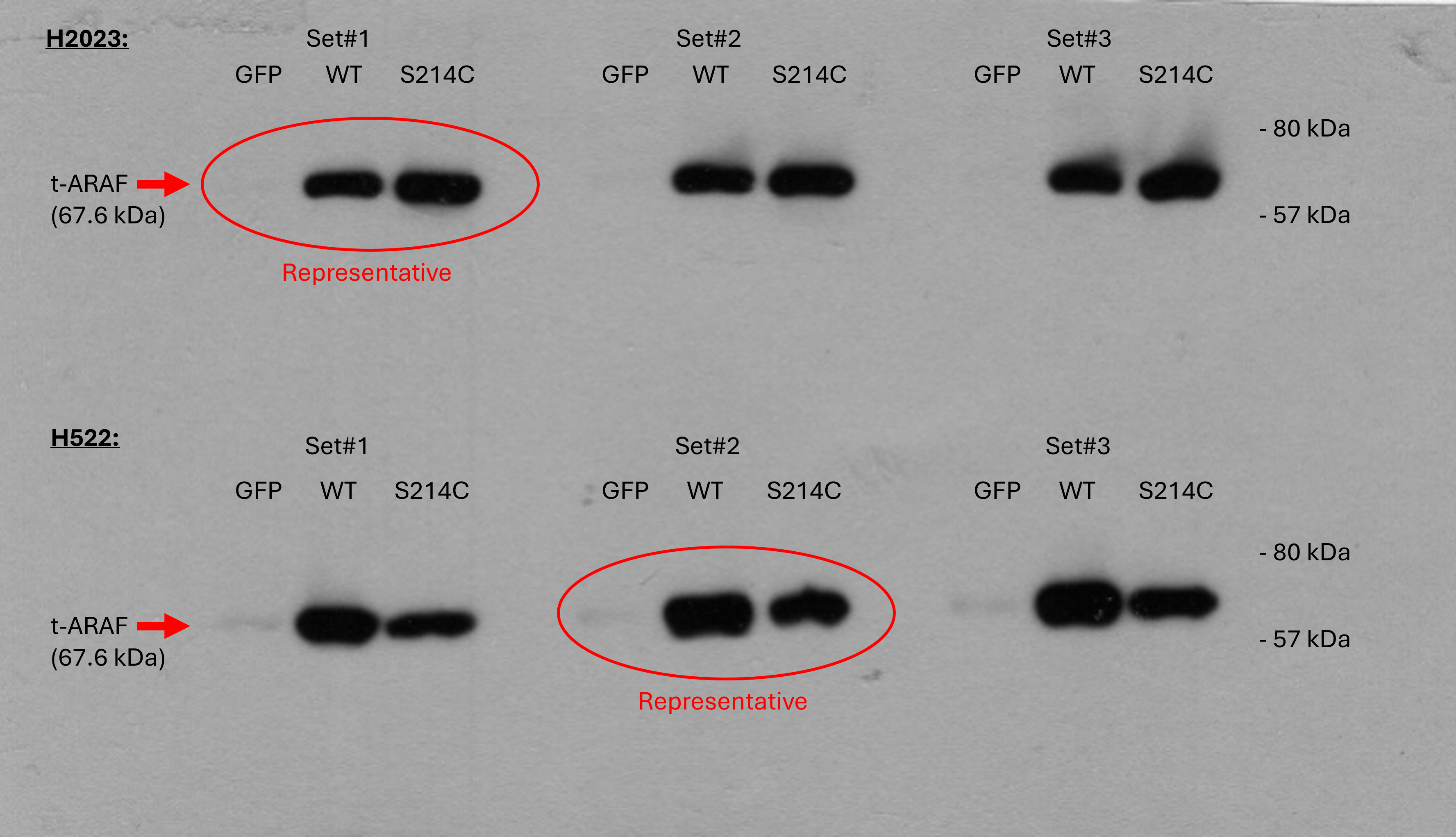

Supplement: Supplementary file 1 [file cancers-17-02246-s001.zip › Western blot_Raw Data/WB_Figure 2A_t-ARAF.tif]

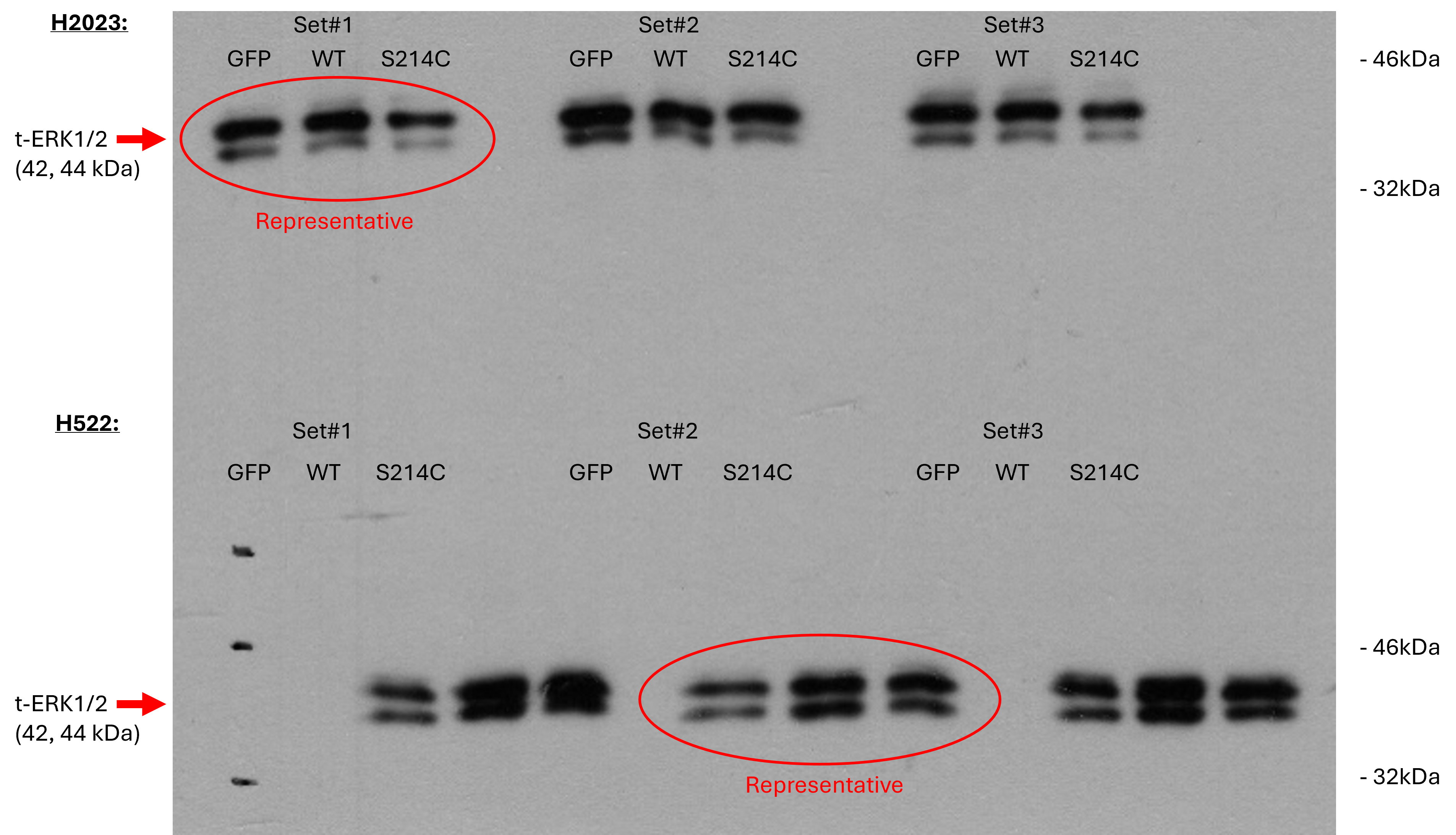

Supplement: Supplementary file 1 [file cancers-17-02246-s001.zip › Western blot_Raw Data/WB_Figure 2A_t-ERK.tif]

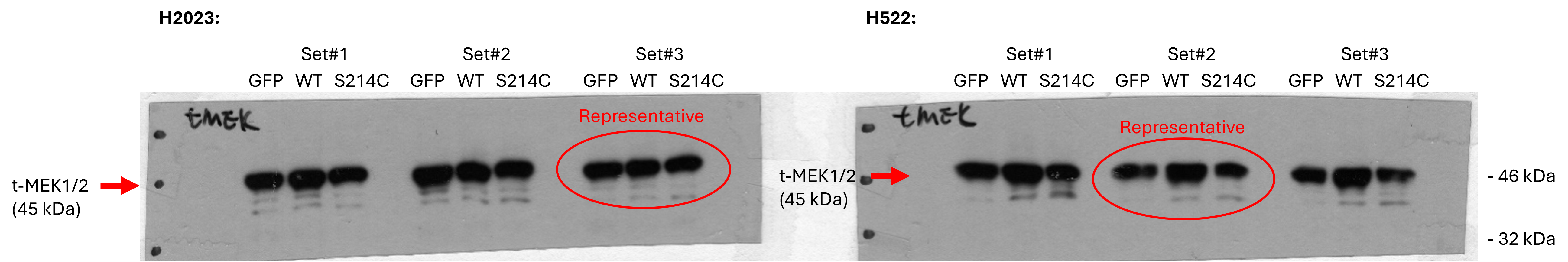

Supplement: Supplementary file 1 [file cancers-17-02246-s001.zip › Western blot_Raw Data/WB_Figure 2A_t-MEK.tif]

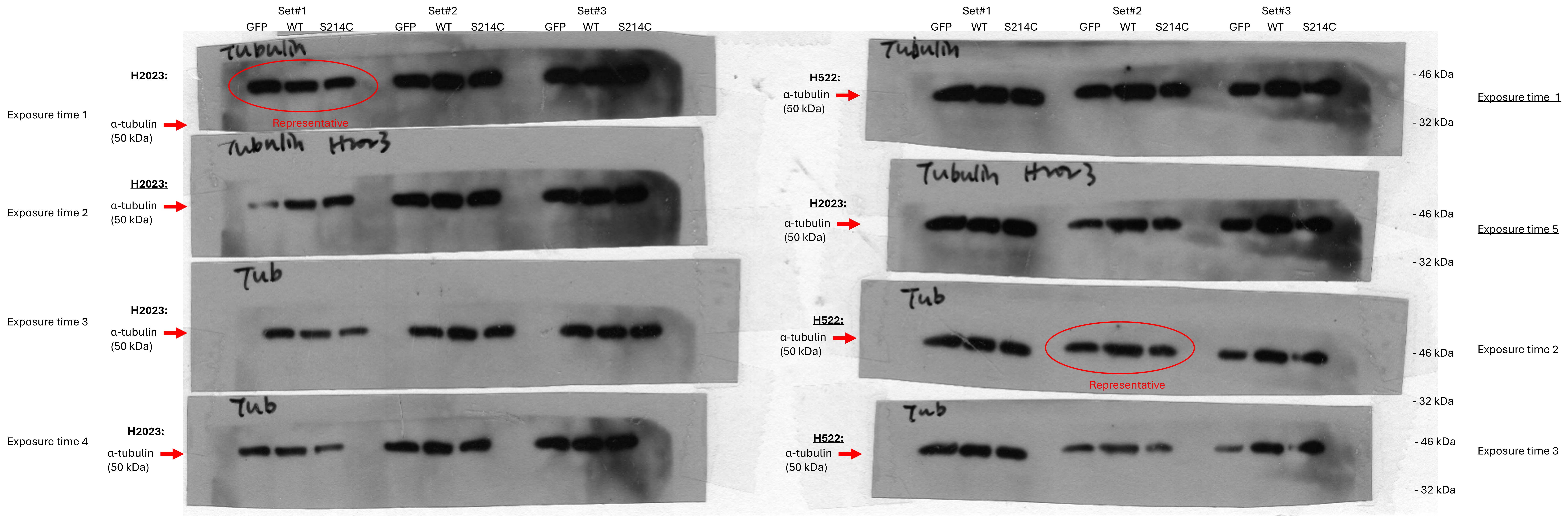

Supplement: Supplementary file 1 [file cancers-17-02246-s001.zip › Western blot_Raw Data/WB_Figure 2A_α-tubulin.tif]

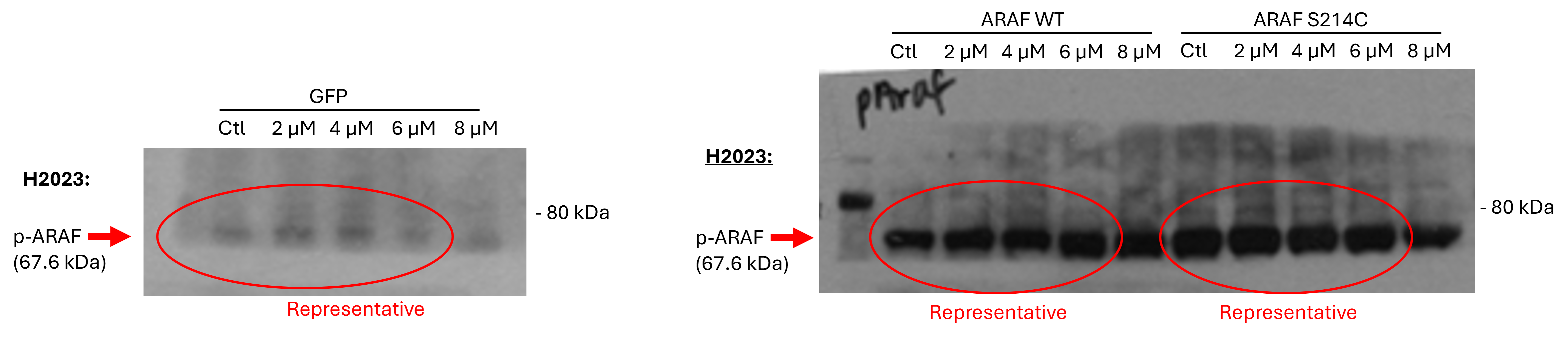

Supplement: Supplementary file 1 [file cancers-17-02246-s001.zip › Western blot_Raw Data/WB_Figure 5F_p-ARAF.tif]

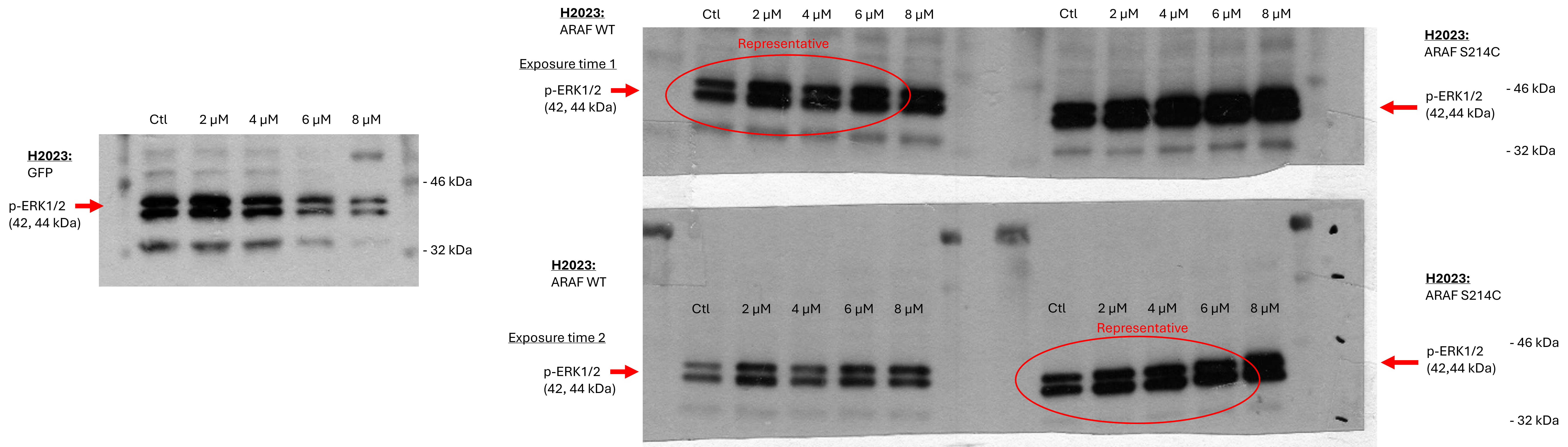

Supplement: Supplementary file 1 [file cancers-17-02246-s001.zip › Western blot_Raw Data/WB_Figure 5F_p-ERK.tif]

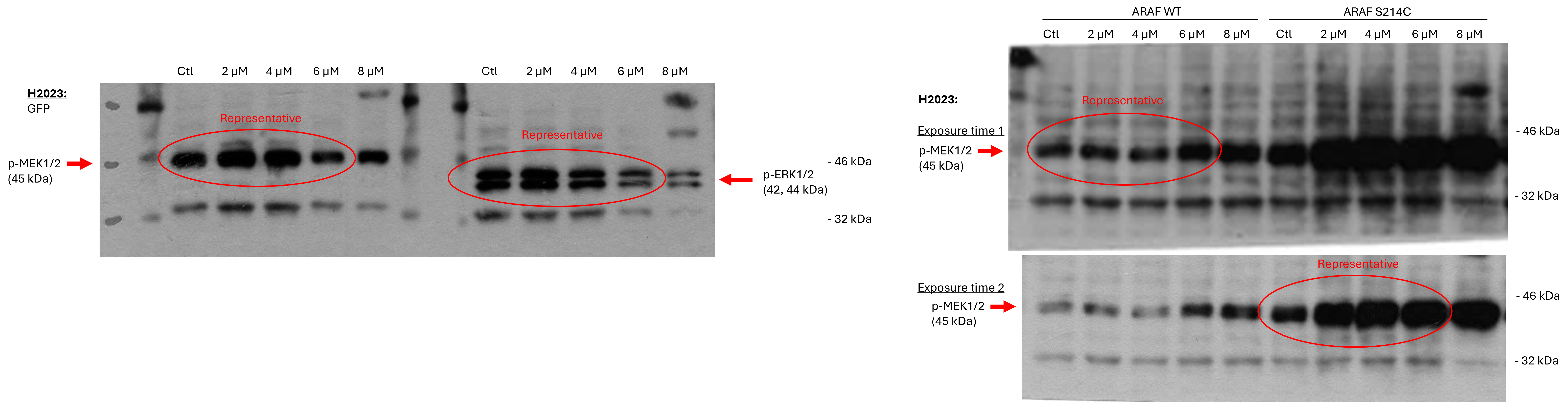

Supplement: Supplementary file 1 [file cancers-17-02246-s001.zip › Western blot_Raw Data/WB_Figure 5F_p-MEK.tif]

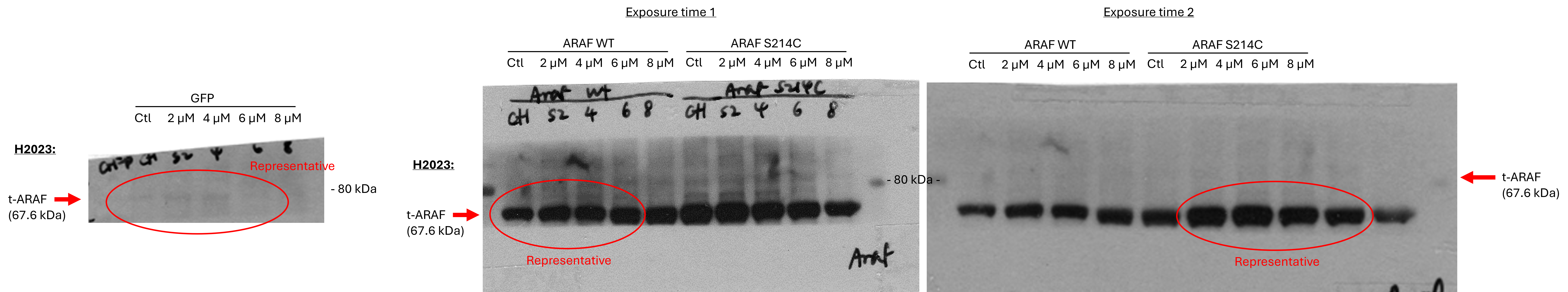

Supplement: Supplementary file 1 [file cancers-17-02246-s001.zip › Western blot_Raw Data/WB_Figure 5F_t-ARAF.tif]

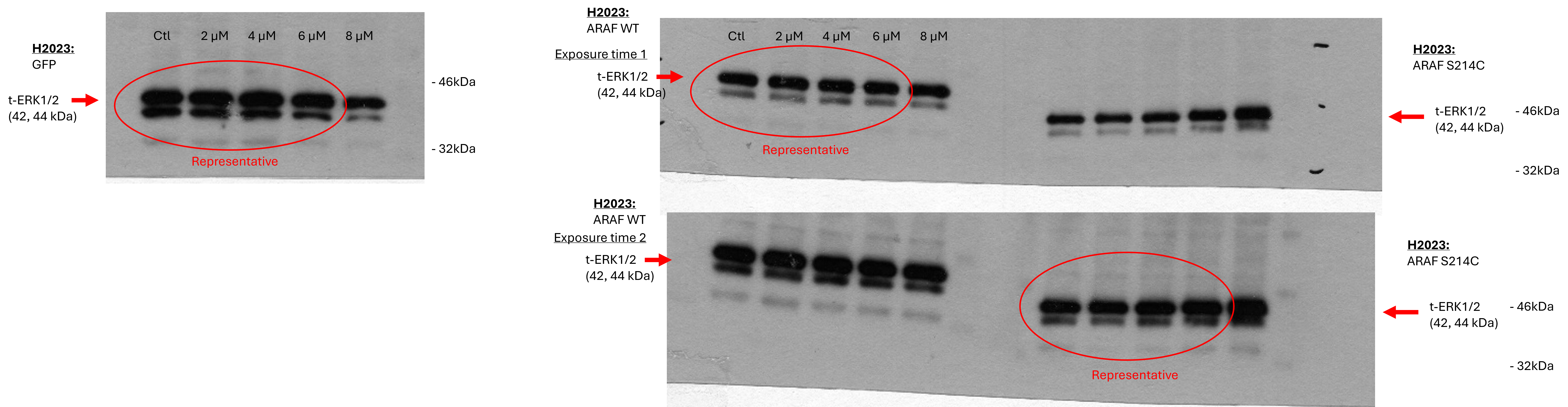

Supplement: Supplementary file 1 [file cancers-17-02246-s001.zip › Western blot_Raw Data/WB_Figure 5F_t-ERK.tif]

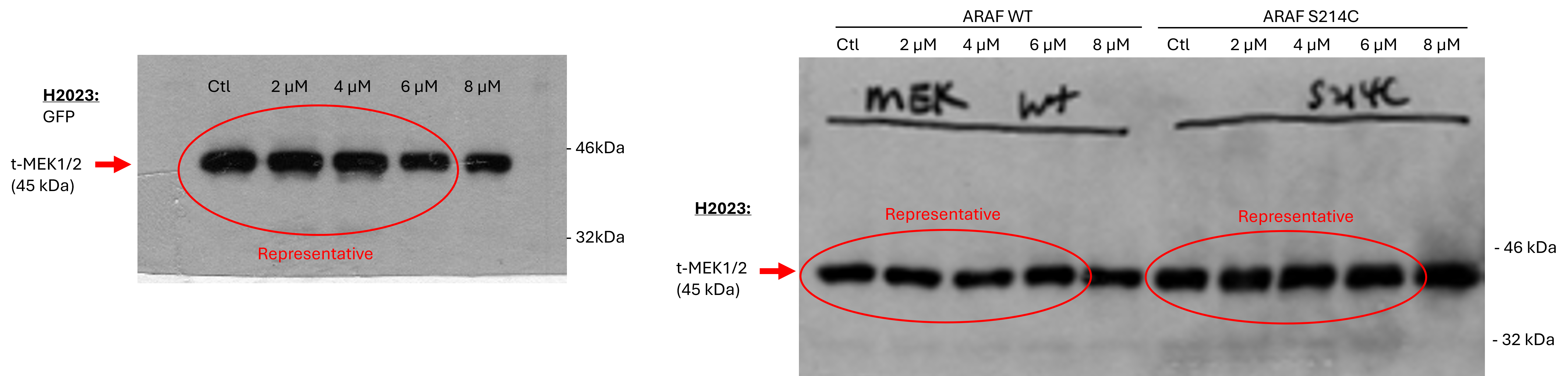

Supplement: Supplementary file 1 [file cancers-17-02246-s001.zip › Western blot_Raw Data/WB_Figure 5F_t-MEK.tif]

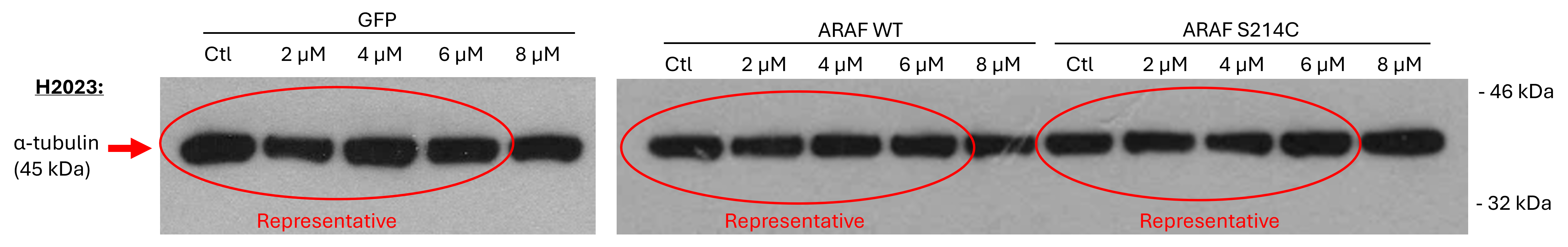

Supplement: Supplementary file 1 [file cancers-17-02246-s001.zip › Western blot_Raw Data/WB_Figure 5F_α-tubulin.tif]
